# Shift of antibiotic resistance after the COVID-19 pandemic: results from the INVIFAR Network

**DOI:** 10.1093/jacamr/dlag129

**Published:** 2026-07-14

**Authors:** Elvira Garza-González, Fabian Rojas-Larios, María del Rosario Vázquez-Larios, Melissa Hernández-Durán, José Manuel Feliciano-Guzman, Christian Daniel Mireles-Davalos, Bernardo Alfonso Martínez-Guerra, Adolfo Gomez-Quiroz, Jesus Alfonso Aguirre-Torres, Juan Pablo Mena-Ramírez, Nicolás Rogelio Eric Barlandas-Rendón, Rogelio de J Treviño-Rangel, Elena Victoria Choy-Chang, Juan Luis Jaime-Sánchez, Enrique Bolado-Martínez, Laura Karina Avilés-Benítez, Carlos Antonio Couoh-May, Eduardo López-Gutiérrez, Juana Hernandez-Martinez, Iris Yazmin Hernandez-Cordova, Aldo Rafael Silva-Gamiño, Katia Cecilia Garcia-Estrada, Joaquín Rincón-Zuno, Mariana Gil-Veloz, Ismelda López-Ovilla, Cecilia Teresita Morales-de-la-Peña, Lilian de Jesús Tirado-Morales, Jonathan Isaac Arauz-Cabrera, Laura Isabel López-Moreno, Guillermo Jacobo-Baca, Carla Rocio Huerta-Baltazar, Lorena Rodríguez-Muñoz, Ana Elizabeth Ortiz-Porcayo, Cecilia Padilla-Ibarra, Alma Denia López-Vázquez, Maricruz Gutiérrez-Brito, Anabel Valenzuela-Oroz, Maria Angelina Quevedo-Ramos, Maribel López-García, Carolina Elizabeth Torres-Barajas, Elizabeth Hernández-Guillén, Filiberto Alejandro Martínez-Lazo, Vanessa Azenet Abigail García-Ortega, Martha Dorado-del-Rio, Alondra Castillo-Jacome, Jesus Eduardo Solis-Hernandez, Manuel G Ballesteros-Monrreal, Zaira Lucero Clemente-Callejas, Rafael Franco-Cendejas, Luis Esaú López-Jácome

**Affiliations:** Departamento de Bioquímica y Medicina Molecular, Facultad de Medicina, Universidad Autónoma de Nuevo León, Monterrey, Mexico; Laboratorio de Microbiología, Facultad de Medicina, Universidad de Colima, Hospital Regional Universitario IMSS Bienestar; Laboratorio de Microbiologia, Servicio de Infectologia y Microbiologia Clínica, Instituto Nacional de Cardiología Ignacio ChávezMexico City, Mexico; Laboratorio de Microbiología Clínica, Instituto Nacional de Rehabilitación Luis Guillermo Ibarra Ibarra Mexico City, Mexico; Laboratorio de Patología Clínica, Hospital de Especialidades Pediátricas IMSS-Bienestar, Tuxtla Gutierrez, Mexico; Laboratorio de Microbiologia Clinica, Instituto Nacional de Enfermedades Respiratorias Ismael Cosio Villegas; Departamento de Infectología, Laboratorio de Microbiología Clínica, Instituto Nacional de Ciencias Médicas y Nutrición Salvador Zubirán, Mexico City, Mexico; Laboratorio de Microbiología, Hospital Civil Fray Antonio Alcalde, Guadalajara, Mexico; Laboratorio de Microbiología, Hospital del Niño Morelense; Laboratorio de Microbiología, Hospital General de Zona N. 21 IMSS Tepatitlán de Morelos, Centro Universitario de los Altos (CUALTOS) Universidad de Guadalajara, Tepatitlán de Morelos, Mexico; Laboratorio de Microbiología Clínica y Farmacéutica, Universidad Autónoma de Guerrero, Chilpancingo, Mexico; Departamento de Microbiología, Facultad de Medicina, Universidad Autónoma de Nuevo León; Laboratorio de Microbiología, Hospital General De Zona No.1 IMSS Nueva Frontera; Laboratorio de Bacteriología, Laboratorio Estatal de Salud Pública, Morelia, Mexico; Departamento de Ciencias Químico-Biológicas, Universidad de Sonora, Hermosillo, Mexico; Laboratorio de Microbiología y Parasitología, Hospital Infantil de Morelia, Morelia, Mexico; laboratorio de Microbiologia, Hospital General Dr. Agustin O'Horan, Merida, Mexico; Laboratorio de Microbiología, Hospital Regional de Alta Especialidad de Oaxaca, San Bartolo Coyotepec, Mexico; Área de Microbiología, Laboratorios Clínicos de Referencia MICROTEC. Mexico City, Mexico; Laboratorio de Microbiología, Hospital Regional de Alta Especialidad Bicentenario de la Independencia, Tultitlan, Mexico; Laboratorio de Microbiología, Hospital Ángeles Morelia, Morelia, Mexico; Laboratorio de Micrbiología Clínica, Laboratorios Preciado Montes, Colima, Mexico; Departamento de Infectología, Hospital para el Niño de Toluca IMIEM, Toluca, Mexico; Servicio de Infectología, Hospital Regional de Alta Especialidad del Bajío, León, Mexico; Área de Bacteriología, Hospital Dr. Jesús Gilberto Gómez Maza, Tuxtla Gutiérrez, Mexico; Área de Bacteriología, Hospital General Juan María de Salvatierra, La Paz, Mexico; Área de Bacteriología, Hospital General Tapachula, Tapachula, Mexico; Departamento de Farmacología, Facultad de Medicina, Universidad Autónoma de Baja California, Mexicali, Mexico; Laboratorio Clinico Área Microbiologia, Hospital Galenia, Cancún, Mexico; Laboratorio de Microbiología, Centro Universitario de Salud, Universidad Autónoma de Nuevo León. Monterrey, Mexico; Laboratorio de Microbiología, Hospital General Morelia, Morelia, Mexico; Departamento de Infectología Hospital del Niño ‘Dr. Federico Gómez Santos, Saltillo, Mexico; Laboratorio Clinico Área Microbiología, Hospital Regional ‘Alta Especialidad’ ISSSTE Monterrey, Monterrey, Mexico; Laboratorio de Microbiología, Hospital General del Estado, Hermosillo, Mexico; Laboratorio de Análisis Clínicos, Centro Integral de Atención a la Salud Unidad Sur ISSSTESON, Hermosillo, Mexico; Laboratorio de Microbiología y Epidemiología, Hospital para la Niñez Poblana. San Andres Cholula, Mexico; Laboratorio de Microbiología, Hospital Adolfo López Mateos, Ciudad Obregón, Mexico; Laboratorio de Microbiologia, Hospital General León, León, Mexico; Área de Bacteriología, Hospital de la Madre y el Niño Guerrerense, Chilpancingo, Mexico; Laboratorio de Microbiología Clínica, Hospital General Silao, Silao, Mexico; Laboratorio Clínico, Área de Microbiología, Hospital General ISSSTE SLP, San Luis Potosí, Mexico; Departamento de Microbiolgía Clínica, BioDiagnostics Laboratorio Clínico, Apizaco, Mexico; Laboratorio Clínico, Área de Microbiología, Christus Muguerza Hospital Faro del Mayab, Mérida, Mexico; Microbiología Clínica, Laboratorio Dorado. Mexicali, Mexico; Laboratorio Clínico, Hospital Español Veracruz, Veracruz, Mexico; Laboratorio Clínico, Lapi Laboratorio Médico, Mexico City, Mexico; Departamento de Ciencias Químico Biológicas y Agropecuarias, Universidad de Sonora, Caborca, Mexico; Laboratorio de Microbiología, Hospital General de Querétaro, Querétaro, Mexico; Laboratorio de Microbiología Clínica, Instituto Nacional de Rehabilitación Luis Guillermo Ibarra Ibarra Mexico City, Mexico; Laboratorio de Microbiología Clínica, Instituto Nacional de Rehabilitación Luis Guillermo Ibarra Ibarra Mexico City, Mexico; Departamento de Biología, Facultad de Química, Universidad Nacional Autónoma de México, Mexico City, Mexico

## Abstract

**Objectives:**

In this study, we evaluated temporal changes in antimicrobial resistance (AMR) before, during and after the COVID-19 pandemic in Mexico.

**Methods:**

We conducted a multicentre, retrospective surveillance study that included clinically significant isolates of Gram-positive and Gram-negative bacteria collected across Mexico between January 2019 and December 2024. Differences in antibiotic resistance among clinical specimen types and across pre-pandemic, pandemic and post-pandemic periods were assessed using Fisher’s exact test and linear regression, as appropriate.

**Results:**

A total of 156 193 isolates were analysed. Among Enterobacterales, extended-spectrum β-lactamase-producing *Escherichia coli* increased from the pre-pandemic to post-pandemic period (46.5%–48.9%, *P* for trend = 0.005). Also, resistance to ertapenem (2.0% to 3.2%) and meropenem (1.2% to 2.7%) increased significantly (both *P* for trend < 0.001). In contrast, the *Acinetobacter baumannii*-*calcoaceticus* complex exhibited a significant post-pandemic decline in resistance to FEP, decreasing from 82.2% in the pre-pandemic period to 48.5% post-pandemic. Similar downward trends were observed for MEM (82.5%–65.1%) and IPM (77.2%–63.9%) (*P* for trend < 0.001). For *Pseudomonas aeruginosa*, ceftazidime resistance increased during the pandemic period (18.8%–24.4%) and declined in the post-pandemic period (20.0%). Among Gram-positive organisms, vancomycin resistance in *Enterococcus faecium* increased in the post-pandemic period (29.1%–43.1%, *P* < 0.001), and in urine isolates, a significant increase in AMP resistance was observed (76.1% in the pre-pandemic to 83.9% in the pandemic to 86.1% in the post-pandemic (*P* for trend = 0.029). For *S. aureus*, oxacillin resistance increased from 13.9% (pre-pandemic) to 19.0% (pandemic) but declined thereafter (16.5%, *P* < 0.001).

**Conclusions:**

The COVID-19 pandemic was a major driver of AMR dynamics in Mexico, and its impact persists. While resistance rates declined for *A. baumannii*, rising ESBL prevalence and increasing fluoroquinolone resistance highlight persistent and emerging threats. Sustained surveillance and strengthened antimicrobial stewardship are essential to mitigate long-term post-pandemic AMR consequences.

## Introduction

Antimicrobial resistance (AMR) is considered a major global health threat, compromising the effective treatment of many infections.^1,2^ Gram-negative pathogens such as *Escherichia coli*, *Klebsiella pneumoniae*, *Enterobacter cloacae* complex, *Pseudomonas aeruginosa* and *Acinetobacter baumannii*, along with Gram-positive organisms such as *Staphylococcus aureus* and *Enterococcus faecium*, exhibit rising rates of resistance to multiple antibiotics.^3–5^ These pathogens are included in the World Health Organization bacterial priority pathogens list for guiding research and investments to address this threat.^6^

The coronavirus disease 2019 (COVID-19) pandemic significantly impacted the use of antibiotics and the surveillance of AMR systems.^7–9^ Due to the pandemic-driven changes in healthcare workflows, increased use of broad-spectrum antibiotics and prolonged hospitalizations, shifts in resistance patterns were observed worldwide, exacerbating AMR.^10,11,12^ However, the behaviour of AMR during the post-pandemic period remains poorly understood.

The INVIFAR Network (Thematic Network for Research and Surveillance of Antimicrobial Resistance, by its acronym in Spanish) has enabled the systematic collection, analysis and dissemination of resistance data from key bacterial pathogens of clinical importance.^13–18^ The aim of this study was to describe changes in the resistance profiles of different pathogens through a multicentre, longitudinal analysis including the pre-pandemic, pandemic and post-pandemic periods (2019–24).

## Methods

### Study design

In this multicentre, retrospective surveillance study, AMR patterns from clinically relevant Gram-negative and Gram-positive isolates collected between 1 January 2019 and 31 December 2024 were evaluated. Data were divided into three periods: pre-pandemic (1 January 2019–23 March 2020), pandemic (24 March 2020–4 May 2023), and post-pandemic (5 May 2023–31 December 2024).

### Bacterial identification, antimicrobial susceptibility

Data from microbiologically significant isolates of *E. coli*, *K. pneumoniae*, *Enterobacter cloacae* complex, *A. baumannii*, *P. aeruginosa*, *S. aureus*, and *E. faecium* were included. Clinical isolates recovered from blood, urine, lower respiratory tract (LRT) specimens (excluding sputum), abscesses and biopsies and cerebrospinal fluid (CSF) were included. Bacterial identification and antimicrobial susceptibility testing (AST) were performed at each participating centre using locally available methods. Antibiotics included were amoxicillin/clavulanic acid (AMC), amikacin (AMK), ampicillin (AMP), ceftazidime (CAZ), ciprofloxacin (CIP), clindamycin (CLI), ceftriaxone (CRO), cefotaxime (CTX), ceftazidime/avibactam (CZA), ceftolozane/tazobactam (CZT), doripenem (DOR), erythromycin (ERY), ertapenem (ETP), cefepime (FEP), cefoxitin (FOX), high-dose gentamicin (GEH), gentamicin (GEN), imipenem (IPM), linezolid (LNZ), levofloxacin (LVX), meropenem (MEM), nitrofurantoin (NIT), norfloxacin (NOR), oxacillin (OXA), penicillin (PEN), rifampicin (RIF), ampicillin/sulbactam (SAM), sulfamethoxazole/trimethoprim (SXT), tetracycline (TCY), piperacillin/tazobactam (TZP) and vancomycin (VAN). All susceptibility interpretations followed the Clinical and Laboratory Standards Institute (CLSI) M100-S35 (2025) breakpoints.^19^ In addition, the presence of extended-spectrum β-lactamases (ESBL) was documented.

### Data management and analysis

Raw data were directly exported from the automated platforms and subjected to quality review. Only identification results with concordance values >95% were included. The curated datasets were analysed using the Backlink tool and WHONET 2025 software (https://whonet.org/). This software was used to select only the first isolate per patient.^20^

Resistance rates for each bacterial species were compared between clinical specimens and across study time periods. Only organisms with at least 30 AST-tested isolates were included in categorical comparisons.^20^ Differences in resistance frequencies between study periods and clinical specimens were assessed using Fisher’s exact test. To explore changes over time, linear regression models were applied to evaluate the magnitude and direction of temporal trends. A two-tailed *P* < 0.05 was considered statistically significant. Additionally, absolute differences in resistance proportions (Δ%), odds ratios (ORs) and 95% confidence intervals (CIs) were calculated. A two-sided *P* < 0.05 was considered statistically significant.

### Ethical considerations

This study received ethical approval from the Research Committee of the Instituto Nacional de Rehabilitación Luis Guillermo Ibarra Ibarra (Approval No. 55/22 AC). The need for informed consent was waived because the dataset consisted of anonymized surveillance results. All study procedures conformed to the ethical principles outlined in the Declaration of Helsinki.

## Results

### Participating centres and microbiological data

Data were collected from 47 participating centres across 23 federal entities (Table S1, available as Supplementary data at *JAC-AMR* Online). Bacterial identification was predominantly performed using automated systems, including VITEK-2 (*n* = 32), MicroScan WalkAway (*n* = 5), BD Phoenix (*n* = 2) and MALDI-TOF mass spectrometry (*n* = 2); additionally, three centres used conventional biochemical methods. Three centres reported the use of combined identification platforms (VITEK-2 and BD Phoenix, MALDI-TOF and VITEK-2, and MALDI-TOF and BD Phoenix).

AST was mainly conducted using automated systems, including VITEK-2 (*n* = 34), MicroScan WalkAway (*n* = 5) and BD Phoenix (*n* = 3), while three centres used the Kirby–Bauer method. Two centres employed multiple AST platforms (VITEK-2 and BD Phoenix and VITEK-2 and Sensititre).

### Drug resistance in Gram-negative and Gram-positive by clinical specimens

A total of 156 193 isolates were included across all clinical specimens (*E. coli*, *n* = 78 212; *K. pneumoniae*, *n* = 21 055; *E. cloacae*, *n* = 7139; *A. baumannii*, *n* = 5943; *P. aeruginosa*, *n* = 21 078; *S. aureus*, *n* = 19 101; and *E. faecium*, *n* = 3665).

**Figure 1.**
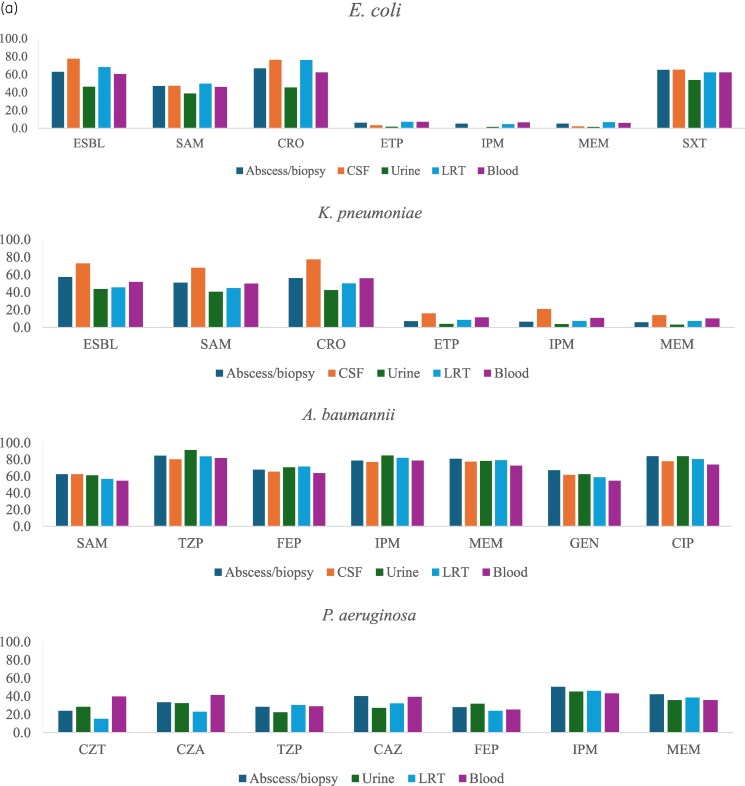

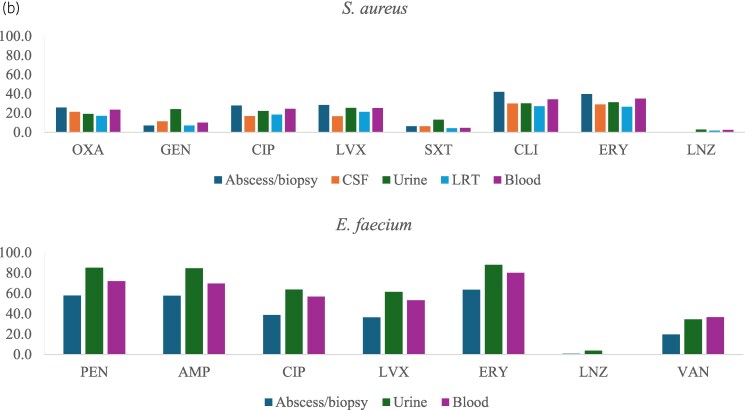
A. Distribution of antibiotic resistance by clinical specimen in Gram-negative bacteria. ESBL, extended-spectrum β-lactamase; SAM, ampicillin/sulbactam; CRO, ceftriaxone; ETP, ertapenem; IPM, imipenem; MEM, meropenem; SXT, sulfamethoxazole/trimethoprim; TZP, piperacillin/tazobactam; CZT, ceftolozane/tazobactam; CZA, ceftazidime/avibactam; CAZ, ceftazidime; FEP, cefepime; GEN, gentamicin; CIP, ciprofloxacin; CSF, cerebrospinal fluid; LRT, lower respiratory tract. B. Distribution of antibiotic resistance by clinical specimen in Gram-positive organisms. OXA, oxacillin; GEN, gentamicin; CIP, ciprofloxacin; LVX, levofloxacin; SXT, sulfamethoxazole/trimethoprim; CLI, clindamycin; ERY, erythromycin; LNZ, linezolid; PEN, penicillin; AMP, ampicillin; ; VAN, vancomycin; CSF, cerebrospinal fluid; LRT, lower respiratory tract.

For all organisms, significant differences in resistance profiles were observed by specimen type (*P* < 0.001 unless otherwise indicated) (Table S2, Figure 1a, Figure 1b).

The highest proportion of ESBL-producing *E. coli* (77.3%) and ESBL-producing *K. pneumoniae* (73.0%) was detected in CSF. For *E. coli*, the resistance rates to carbapenems were below 7.1% for ETP, IPM and MEM. In *K. pneumoniae*, carbapenem resistance remained consistently higher in CSF isolates (16.0%–21.1% for ETP and IPM, respectively) compared with other specimen types. It should be noted that the sample size for CSF isolates was small; therefore, these findings should be interpreted with caution.

For *E. cloacae*, resistance to carbapenems was variable, with ETP resistance ranging from 7.2% to 15.9% and IPM resistance from 2.4% to 10.1%. All comparisons were statistically significant.

*A. baumannii* exhibited a high resistance rate across most antibiotics, particularly to carbapenems (up to 84.8% resistant). Differences across specimen types were significant for all drugs (*P* < 0.003), except for IPM (*P* = 0.075). For *P. aeruginosa*, CZT resistance ranged from 15.2% (LRT) to 39.9% (blood). CZA resistance ranged from 23.1% (LRT) to 41.4% (blood). IPM resistance ranged from 43.2% (blood) to 50.5% (abscess/biopsy), and MEM resistance ranged from 36.0% (blood and urine) to 42.4% (abscess/biopsy). All comparisons showed significant differences across specimens.

For *S. aureus*, OXA resistance was highest in abscess/biopsy isolates (25.8%) and lowest in LRT (17.0%). Fluoroquinolone resistance (CIP and LVX) was highest in abscess/biopsy isolates (27.8%–28.5%), and LNZ susceptibility remained high (≥97%) in all groups. SXT resistance was higher in urine clinical isolates (12.9%) (*P* < 0.001), while CLI resistance was higher in isolates from abscesses and biopsy specimens (42%) (*P* < 0.001).

For *E. faecium*, high resistance to PEN and AMP was observed, especially in urine isolates (85.2% and 84.5%, respectively). VAN resistance ranged from 19.5% (abscess/biopsy) to 36.6% (blood). All differences were significant.

### Drug resistance in Gram-negative and Gram-positive by study period

For *E. coli*, an increase in ESBL-producing *E. coli* was observed from the pre-pandemic (46.5%) to the post-pandemic period (48.9%) (*P* = 0.018; *P* for trend = 0.005); meanwhile, for *K. pneumoniae*, no significant differences in ESBL frequency were detected.

**Figure 2.**
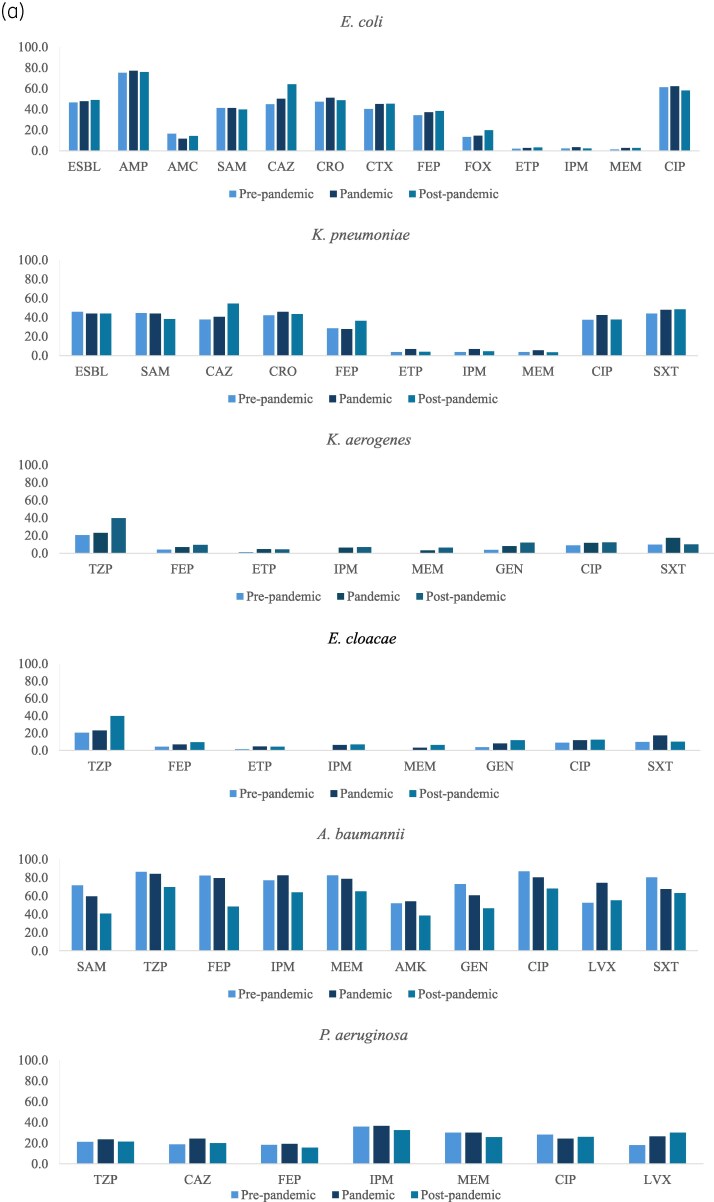

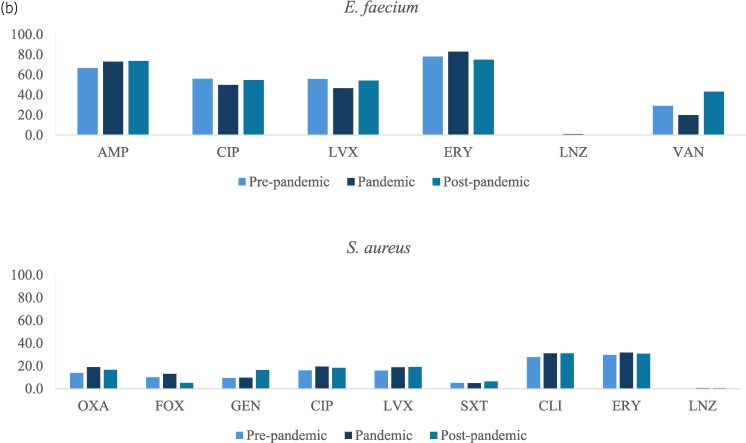
A. Distribution of antibiotic resistance by period in Gram negatives. ESBL, extended-spectrum β-lactamase; AMP, ampicillin; AMC, amoxicillin/clavulanic acid; SAM, ampicillin/sulbactam; CAZ, ceftazidime; CRO, ceftriaxone; CTX, cefotaxime; FEP, cefepime; FOX, cefoxitin; ETP, ertapenem; IPM, imipenem; MEM, meropenem; CIP, ciprofloxacin; SXT, sulfamethoxazole/trimethoprim; TZP, piperacillin/tazobactam; GEN, gentamicin; LVX, levofloxacin. B. Distribution of antibiotic resistance by period in Gram-positive bacteria. AMP, ampicillin; CIP, ciprofloxacin; LVX, levofloxacin; ERY, erythromycin; LNZ, linezolid; VAN, vancomycin; OXA, oxacillin; FOX, cefoxitin; GEN, gentamicin; SXT, sulfamethoxazole/trimethoprim; CLI, clindamycin.

For *E. coli*, a significant increase in resistance was observed for CAZ (from 44.7% to 64.0%), CTX (from 40.4% to 45.3%) and FEP (34.1% to 38.4%) (all *P* for trend < 0.001), whereas a significant decrease was observed for CIP (from 61.3% to 58.1%) (Table S3, Table S5, Figure 2a). In contrast, the trend for CRO was not statistically significant. Carbapenem resistance increased for ETP (2.0%–3.2%) and MEM (1.2%–2.7%) (both *P* for trend < 0.001).

For *K. pneumoniae*, resistance to CAZ and FEP increased in the post-pandemic period (37.8%–54.4% and 28.5%–36.3%, respectively; both *P* for trend < 0.001). No significant trends were detected for CIP and CRO. Resistance to ERT increased from 3.8% (pre-pandemic) to 6.9% (pandemic) and decreased to 4.1% (post-pandemic). Although the overall *P*-value was significant (*P* < 0.001), the trend was not statistically significant (*P* for trend = 0.428).

For *E. cloacae* complex, significant increases in resistance were observed for FEP (5.1%–10.1%), MEM (2.4%–5.2%) and CIP (7.6%–15.4%) across study periods (all *P* for trend <  0.001).

For *A. baumannii*, resistance remained high across all β-lactams and fluoroquinolones but showed a significant decline in the post-pandemic period. FEP resistance decreased from a pre-pandemic 82.2% to a post-pandemic 48.5%. Similar trends were observed for MEM (82.5%–65.1%) and IPM (77.2%–63.9%) (*P* for trend < 0.001).

Aminoglycoside susceptibility improved, with GEN resistance declining from 72.9% to 46.5%.

For *P. aeruginosa*, CAZ resistance increased during the pandemic (18.8%–24.4%) but again declined during the post-pandemic period (20.0%). FEP resistance significantly declined (18.2%–15.6%; *P* for trend = 0.017). IPM resistance decreased in the post-pandemic period (35.8%–32.4%), though not significantly.

For *E. faecium*, AMP resistance increased from 66.6% to 73.6% (*P* for trend = 0.022), ERY resistance decreased (78.0% to 74.9%; *P* for trend = 0.006), and VAN resistance increased in the post-pandemic period (29.1%–43.1%) (*P* < 0.001).

For *S. aureus*, OXA resistance increased from 13.9% pre-pandemic to 19.0% during the pandemic period but declined thereafter (16.5%). GEN resistance rose steadily (9.5% to 16.4%; *P* for trend < 0.001). SXT resistance increased slightly from 5.1% in the pre-pandemic period to 6.4% in the post-pandemic period (*P* for trend = 0.028). CLI resistance increased from 27.8% to 31.1% between the pandemic and post-pandemic periods; however, the trend was not statistically significant.

### Drug resistance by period and by clinical specimens

#### Bloodstream isolates

**Figure 3.**
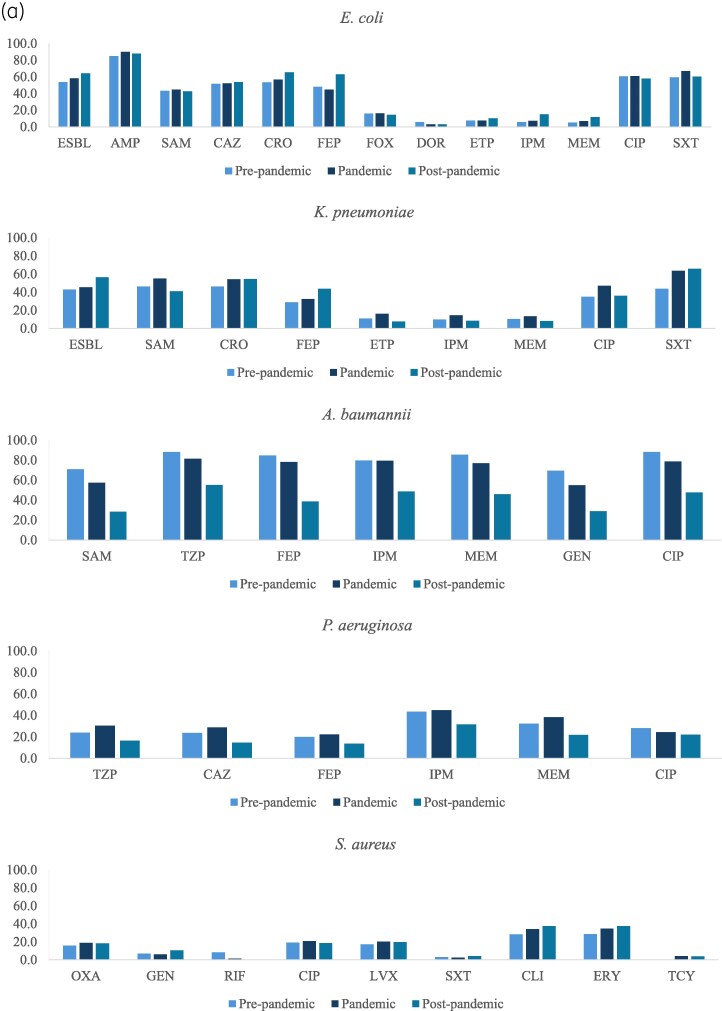

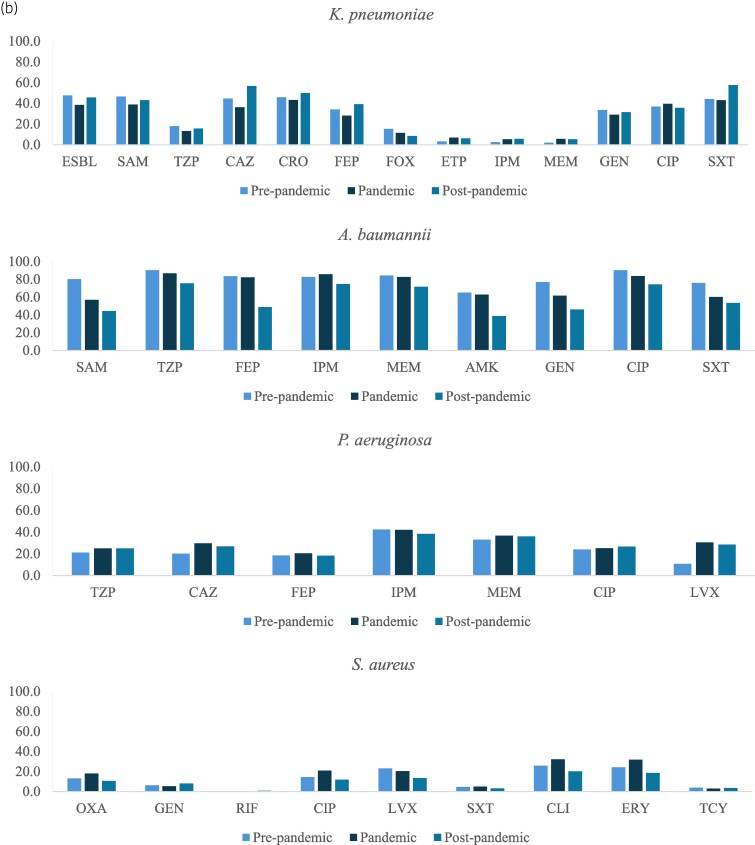

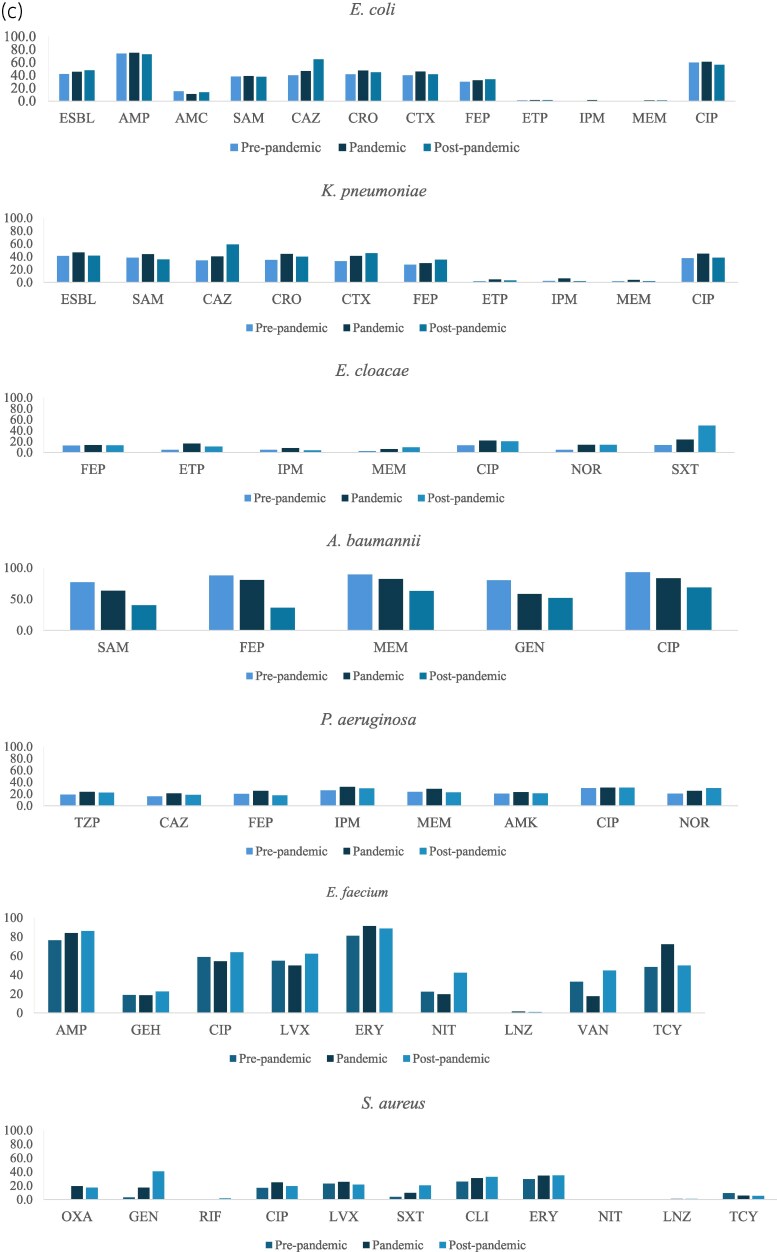

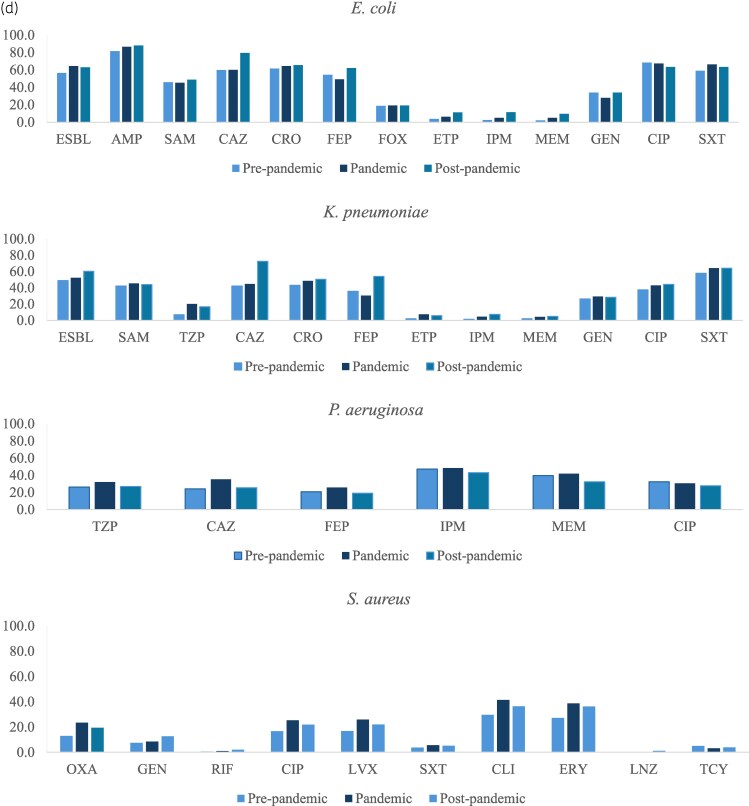
A. Distribution of antibiotic resistance by period in clinical isolates collected from blood. ESBL, extended-spectrum β-lactamase; AMP, ampicillin; SAM, ampicillin/sulbactam; CAZ, ceftazidime; CRO, ceftriaxone; FEP, cefepime; DOR, doripenem; ETP, ertapenem; IPM, imipenem; MEM, meropenem; CIP, ciprofloxacin; SXT, sulfamethoxazole/trimethoprim; TZP, piperacillin/tazobactam; GEN, gentamicin; OXA, oxacillin; RIF, rifampicin; CLI, clindamycin; ERY, erythromycin; TCY, tetracycline; LVX, levofloxacin; FOX, cefoxitin. B. Distribution of antibiotic resistance by period in clinical isolates collected from lower respiratory tract specimens. ESBL, extended-spectrum β-lactamase; SAM, ampicillin/sulbactam; TZP, piperacillin/tazobactam; CAZ, ceftazidime; CRO, ceftriaxone; FEP, cefepime; FOX, cefoxitin; ETP, ertapenem; IPM, imipenem; MEM, meropenem; GEN, gentamicin; CIP, ciprofloxacin; SXT, sulfamethoxazole/trimethoprim; AMK, amikacin; LVX, levofloxacin; OXA, oxacillin; RIF, rifampicin; CLI, clindamycin; ERY, erythromycin; TCY, tetracycline. C. Distribution of antibiotic resistance by period in clinical isolates collected from urine. ESBL, extended-spectrum β-lactamase; AMP, ampicillin; AMC, amoxicillin/clavulanic acid; SAM, ampicillin/sulbactam; CAZ, ceftazidime; CRO, ceftriaxone; CTX, cefotaxime; FEP, cefepime; ETP, ertapenem; IPM, imipenem; MEM, meropenem; CIP, ciprofloxacin; NOR, norfloxacin; SXT, sulfamethoxazole/trimethoprim; AMK, amikacin; GEH, high-dose gentamicin; LVX, levofloxacin; ERY, erythromycin; NIT, nitrofurantoin; LNZ, linezolid; VAN, vancomycin; TCY, tetracycline; OXA, oxacillin; RIF, rifampicin; CLI, clindamycin; GEN, gentamicin. D. Distribution of antibiotic resistance by period in clinical isolates collected from biopsies and abscesses. ESBL, extended-spectrum β-lactamase; AMP, ampicillin; SAM, ampicillin/sulbactam; CAZ, ceftazidime; CRO, ceftriaxone; FEP, cefepime; FOX, cefoxitin; ETP, ertapenem; IPM, imipenem; MEM, meropenem; GEN, gentamicin; CIP, ciprofloxacin; SXT, sulfamethoxazole/trimethoprim; TZP, piperacillin/tazobactam; OXA, oxacillin; RIF, rifampicin; LVX, levofloxacin; CLI, clindamycin; ERY, erythromycin; LNZ, linezolid; TCY, tetracycline.

Among *E. coli* bloodstream isolates, the proportion of ESBL producers increased progressively across the study periods (53.8% pre-pandemic, 58.2% pandemic and 64.1% post-pandemic; *P* for trend = 0.022). ESBL positivity in *K. pneumoniae* increased from 43.0% (pre-pandemic) to 45.3% (pandemic) and to 56.4% (post-pandemic) (*P* for trend = 0.010) (Table S4A, Table S6, Figure 3a).

For *E. coli*, a significant increase in resistance was observed for FEP (47.91%–62.9%) (*P* for trend < 0.001), and CIP resistance changed from 60.6% in the pre-pandemic period to 60.8% during the pandemic and decreased to 57.9% in the post-pandemic period (*P* for trend < 0.001). Carbapenem resistance increased steadily for ETP (7.4%–10.2%) and MEM (5.1%–11.8%) (both *P* for trend < 0.001).

For *K. pneumoniae*, significant increases in resistance were observed for FEP (28.9% to 44.0%) (both *P* < 0.001). In contrast, no significant difference was observed for CRO (*P* for trend = 0.076). Carbapenem resistance fluctuated, with a transient increase during the pandemic but no significant trends. For *E. cloacae*, FEP resistance was from 1.4% to 7.0%, while resistance to MEM (1.3% to 4.3%) and ETP (2.7% to 6.5%) did not increase.

For *A. baumannii*, resistance to most agents was high during the pre-pandemic period but declined significantly in the subsequent periods: MEM from 85.4% to 45.9% and IPM from 79.5% to 48.7% (*P* and *P* for trend < 0.001).

For *P. aeruginosa*, meropenem resistance showed a downward trend post-pandemic (32.4%–21.8%, *P* for trend = 0.002).

AMP resistance in *E. faecium* increased (63.9%–82.9%; *P* for trend = 0.004), and VAN resistance increased from 32.8% during the pandemic to 58.8% post-pandemic (*P* < 0.001).

For *S. aureus*, LNZ susceptibility remained high (≥99%) in all periods. No significant changes were observed for SXT (3.1% versus 2.5% versus 4.0%; *P* = 0.344; *P* for trend = 0.343). In contrast, CLI resistance increased from 28.4% in the pre-pandemic period to 34.0% during the pandemic and 37.4% in the post-pandemic period (*P* for trend = 0.021).

#### Lower respiratory tract isolates

The proportion of ESBL-producing *K. pneumoniae* fluctuated across periods (47.5% pre-pandemic, 38.4% pandemic, 45.7% post-pandemic; *P* = 0.047), but without a significant linear trend (*P* = 0.953) (Table S4B, Table S7, Figure 3b). CAZ resistance decreased during the pandemic (44.6%–36.2%) but increased significantly during the post-pandemic period to 56.7% (*P* < 0.001; *P* for trend = 0.015). Similarly, FEP resistance initially decreased (34.1%–28.1%), followed by a rise to 39.2% post-pandemic period (*P* < 0.001; *P* for trend = 0.026). No statistically significant changes were observed for CRO, ETP, IPM and MEM.

For *E. cloacae* complex, FEP resistance rose sharply from 3.6% pre-pandemic to 12.1% post-pandemic (both *P* and *P* for trend < 0.001). GEN resistance increased (3.6%–18.9%, *P* < 0.001; *P* for trend < 0.001), as did CIP resistance (2.9%–10.1%, *P* < 0.001).

For *A. baumannii*, resistance to most antibiotics was high during the pre-pandemic period but declined over time. Significant reductions were observed for FEP (83.6%–48.6%, *P* < 0.001), MEM (84.4%–71.7%, *P* = 0.013), GEN (77.0%–46.2%, *P* < 0.001) and CIP (90.1%–74.3%, *P* < 0.001). These reductions were consistently supported by significant trend analyses (e.g. FEP and GEN *P* for trend < 0.001). Despite improvements, post-pandemic resistance levels remained high (≥45%) for most agents. For *P. aeruginosa*, resistance rates to most antibiotics remained stable, especially for TZP, CAZ, FEP, MEM and CIP, which exhibited no significant changes. In contrast, LVX resistance increased significantly (10.8%–30.3% during the pandemic, *P* = 0.001; *P* for trend = 0.011) before stabilizing during the post-pandemic period, representing the most notable shift in this species.

OXA resistance in *S. aureus* rose during the pandemic (13.1%–18.1%) but declined to 10.4% in the post-pandemic period (*P* = 0.002). Fluoroquinolone resistance also fluctuated, with CIP resistance peaking at 20.8% in the pandemic period (*P* = 0.001). LVX resistance decreased over time (23.1%–13.3%; *P* for trend = 0.011). No significant changes were observed for SXT (4.5% versus 4.9% versus 3.0%; *P* = 0.383). In contrast, CLI resistance changed from 25.8% in the pre-pandemic period to 32.2% during the pandemic and decreased to 20.2% in the post-pandemic period (*P* < 0.001; *P* for trend = 0.018).

#### Urine isolates

Among *E. coli* urine isolates, the prevalence of ESBL producers increased across periods, rising from 41.9% pre-pandemic to 45.1% during the pandemic and 47.7% post-pandemic (*P* < 0.001; *P* for trend = 0.005) (Table S4C, Table S8, Figure 3c). Among *K. pneumoniae* isolates, the proportion of ESBL-producing isolates increased from 40.9% pre-pandemic to 46.5% pandemic and then decreased to 41.4% during the post-pandemic period (*P* = 0.061).

For *E. coli*, resistance to CAZ, CRO and CTX increased significantly during the pandemic, with CAZ resistance reaching 64.8% post-pandemic (*P* < 0.001; *P* for trend < 0.001). FEP resistance also rose over time (from 29.7% to 32.2% and to 33.6%) (*P* < 0.001), and CIP resistance changed from 59.8% (pre-pandemic) to 60.7% (pandemic) and decreased to 56.4% (post-pandemic) (*P* for trend < 0.001). Carbapenem resistance increased significantly during the pandemic (up to 1.6%) for ETP, IPM and MEM (*P* < 0.001).

For *K. pneumoniae*, a marked rise in CAZ resistance was observed (from 33.8% pre-pandemic to 58.9% post-pandemic, *P* < 0.001; *P* for trend < 0.001). For *K. pneumoniae* isolates recovered from urine, no significant temporal trend in resistance was identified (*P* for trend = 0.234).

Carbapenem resistance showed a fluctuating pattern: ETP resistance rose significantly during the pandemic (4.5%) before declining post-pandemic (2.8%) (*P* = 0.002). Resistance to IPM and MEM followed a similar pattern. For *P. aeruginosa*, no significant temporal differences were observed for TZP, CAZ or CIP. FEP and MEM showed significant increases during the pandemic (from 19.7% to 25.1%, *P* = 0.006, and from 23.5% to 28.3%, *P* = 0.035, respectively), though no significant linear trends were detected.

For *E. faecium*, VAN resistance decreased during the pandemic (17.7%) and then increased to 44.3% during the post-pandemic period (*P* < 0.001; *P* for trend = 0.002), while resistance to LNZ remained low and stable, and no trends were observed for GEH.

Additionally, a significant increase in AMP resistance was observed for urine isolates, rising from 76.1% in the pre-pandemic period to 83.9% during the pandemic and 86.1% in the post-pandemic period (*P* for trend = 0.029).

#### Abscess and biopsy isolates

Among *E. coli* and *K. pneumoniae* isolates, the frequency of ESBL producers across time was not statistically different.

For *E. coli*, CAZ resistance increased from 59.9% pre-pandemic to 79.6% post-pandemic (*P* < 0.001, *P* for trend < 0.001), and similar patterns were observed for FEP (54.5% to 62.3%; *P* < 0.001) (Table S4D, Table S9, Figure 3d). No significant temporal trends in CRO resistance were observed (*P* for trend = 0.103). Carbapenem resistance showed a significant increase for ETP, IPM and MEM across periods (up to 11.4% for post-pandemic) (all *P* < 0.001; *P* for trend < 0.001).

For *K. pneumoniae*, resistance to CAZ increased from 42.3% pre-pandemic to 72.6% post-pandemic (*P* < 0.001). FEP resistance also increased (35.7%–54.0%; *P* < 0.001; *P* for trend < 0.001). No significant temporal trends in CRO resistance were observed (*P* for trend = 0.120). Carbapenem resistance rose during the pandemic, most notably for ETP (7.4%). For *P. aeruginosa*, TZP and CAZ resistance increased during the pandemic (32.1% and 35.3%, respectively) (*P* = 0.028 and *P* = 0.011, respectively), before returning to near-pre-pandemic levels for the post-pandemic period. MEM resistance decreased in the post-pandemic period (from 41.8% to 32.3%; *P* = 0.034; *P* for trend = 0.053).

For *E. faecium*, VAN resistance exhibited a significant and marked increase, rising from 19.2% pre-pandemic to a post-pandemic 41.9% (*P* < 0.001; *P* for trend < 0.001).

OXA resistance in *S. aureus* increased during the pandemic (12.8%–23.5%; *P* = 0.004) and then declined during the post-pandemic period. CLI and ERY resistance both increased significantly during the pandemic (41.3% and 38.7%, respectively) (*P* = 0.009 for each).

SXT resistance did not change significantly across periods (3.7% versus 5.7% versus 5.3%; *P* = 0.553; *P* for trend = 0.529).

## Discussion

The COVID-19 pandemic appears to have influenced AMR dynamics in heterogeneous ways. While several studies have reported increases in AMR during the pandemic, others have documented stable or even decreased resistance rates, underscoring the variability of AMR trends across bacterial species, patient populations and healthcare settings.^21–25^ Furthermore, multiple investigations have shown that pandemic-associated increases in AMR persisted beyond the acute phase of COVID-19, suggesting that the pandemic acted as a significant stressor that reshaped resistance dynamics with effects that healthcare systems continue to struggle with.^21–25^ In this multicentre surveillance study spanning 2019 to 2024, resistance rates increased during the pandemic across several pathogen-antimicrobial combinations, occasionally followed by stabilization or partial reversal in the post-pandemic period. These findings suggest that the pandemic functioned as a transient driver of resistance dynamics, with incomplete and organism-specific reversal following the pandemic phase. In our study, the most pronounced post-pandemic decline was observed in *A. baumannii*. Despite high pre-pandemic resistance levels, substantial reductions were detected across β-lactams, carbapenems and fluoroquinolones. Although this pattern contrasts with the global surge in carbapenem-resistant *A. baumannii* reported during the pandemic, it aligns with reports describing post-pandemic declines following the normalization of hospital operations,^26^ maybe associated with strengthened infection-prevention measures, and reduced reliance on broad-spectrum antibiotics after the initial months of the COVID-19 pandemic.^21^

For *A. baumannii*, resistance remained high across all β-lactams and fluoroquinolones but showed a significant decline in the post-pandemic period. FEP resistance decreased from a pre-pandemic 82.2% to a post-pandemic 48.5%. Similar trends were observed for MEM (82.5%–65.1%) and IPM (77.2%–63.9%) (*P* for trend < 0.001. GEN resistance declined from 72.9% to 46.5%.

The observed post-pandemic decline in carbapenem-resistant *A. baumannii* is a notable finding and may be influenced by multiple factors. Potential explanations include a true ecological improvement related to strengthened antimicrobial stewardship and infection control measures, as well as shifts in the underlying patient population, changes in case volume of centres and possible survivorship bias due to reduced admission of critically ill patients in the post-pandemic period. Given the retrospective and aggregated nature of our data, we were not able to systematically evaluate these factors. Then, our results should be interpreted with caution, as they may reflect a combination of epidemiological and healthcare system–related changes rather than a single underlying cause.

Among Enterobacterales, ESBL-producing *E. coli* increased modestly but significantly from the pre- to the post-pandemic period (46.5%–48.9%). Although the magnitude of change was modest, this trend is consistent with previous reports showing an increase in ESBL-producing *E. coli* following the COVID-19 pandemic, including an investigation of bloodstream infections in a tertiary-care setting, where rates rose from 35.93% to 50.63%.^22^ These findings suggest that *E. coli* may be sensitive to pandemic-related selective pressures, underscoring the importance of continued surveillance.

Among studies reporting decreases in antibiotic resistance for certain pathogens, a recent systematic review and meta-analysis assessed the impact of the COVID-19 pandemic on the prevalence of multidrug-resistant (MDR) bacteria. Of 77 full-text articles screened, 28 were included, and a significant reduction in the prevalence of carbapenem-resistant *A. baumannii*, carbapenem-resistant Enterobacterales and carbapenem-resistant *P. aeruginosa* was observed in the pre- versus during/after-COVID-19 periods.^21^ Together, these findings indicate that the impact of COVID-19 on MDR prevalence has been heterogeneous, varying by pathogen, geographic region and healthcare setting.^26^

Other reports have documented increases in AMR during the COVID-19 pandemic. A report from the Centers for Disease Control and Prevention described a 15% pandemic-associated increase in the rate of resistant organisms, including carbapenem-resistant *Acinetobacter*, methicillin-resistant *S. aureus*, carbapenem-resistant Enterobacterales and ESBL-producing organisms.^27^ In addition, a study from a tertiary-care hospital in the Republic of Srpska, which analysed 4718 invasive bacterial isolates collected between 2015 and 2024, reported increased resistance in *K. pneumoniae* to cephalosporins, fluoroquinolones and carbapenems, as well as increased carbapenem resistance in *P. aeruginosa* and *Acinetobacter* spp.^28^

Interestingly, another report found no impact on AMR. In this study, the impact of the COVID-19 pandemic on AMR across healthcare settings was examined in a systematic review and meta-analysis, and no significant effect on AMR was reported. Of 6036 studies identified, 28 met the inclusion criteria. In that analysis, the proportion of infections caused by ESBL-producing organisms was not significantly different during the COVID-19 period, and no significant change was observed in the incidence of carbapenem-resistant Enterobacterales (mainly *E. coli* and *Klebsiella* spp.).^24^

Of particular concern was the post-pandemic increase in vancomycin-resistant *Enterococcus* (VRE) among bloodstream and urine isolates, with resistance rising significantly from 29.1% to 43.1% (*P* < 0.001). This trend is consistent with reports suggesting that pandemic-related changes in healthcare delivery—such as prolonged hospitalization, increased use of invasive devices and expanded exposure to broad-spectrum antibiotics—facilitated VRE dissemination. Contributing factors likely include disruptions in antimicrobial stewardship and infection-prevention practices, higher patient acuity, staffing challenges and conditions such as overcrowding and prolonged hospital stays, all of which may have promoted transmission. Additional influences may include selective pressure from agents such as daptomycin or linezolid and the potential role of clonal spread (e.g. ST80/ST117 lineages, reported in Spain).^29^ As observed for Gram-negative pathogens, reports on VRE trends during the pandemic are heterogeneous. For example, a tertiary university hospital in Greece reported a marked rise in ICU-associated VRE bloodstream infections, increasing from 4 cases in 2020 to 36 in 2021.^30^ In contrast, a reduction in the duration of a VRE outbreak was observed during the COVID-19 lockdown in Denmark, underscoring the context-dependent impact of pandemic-related interventions on VRE transmission.^31^

Variability among studies, including ours, reflects factors such as inappropriate antibiotic use and disruptions to antimicrobial stewardship programmes (30). Inappropriate antibiotic use is a key driver of AMR.^32^ A recent systematic review (December 2019–May 2023) involving 892 312 COVID-19 patients across 173 studies reported high prevalences of carbapenem-resistant organisms (41.0%), methicillin-resistant *S. aureus* (19.9%), ESBL-producing organisms (24.9%) and VRE (22.9%), with consistently high antibiotic use.^23^ Antibiotic consumption data were unavailable in our study, which represents a central constraint on interpretation, as it precludes meaningful inference about the mechanisms underlying the observed resistance trends. Without data on antimicrobial use, it is not possible to determine whether the observed changes reflect true ecological shifts in resistance or are instead driven by variations in selective pressure, differences in patient case-mix or changes in culturing practices during the pandemic. Consequently, the study cannot disentangle whether changes in resistance are attributable to antimicrobial stewardship, infection-prevention measures or external disruptions to healthcare delivery versus artefacts of sampling or population changes. As a result, the findings should be interpreted as descriptive of temporal trends rather than indicative of underlying drivers.

The post-pandemic declines in resistance observed in *A. baumannii* suggest that some pandemic-associated increases may be partially reversible; however, concurrent rises in ESBL-producing organisms, VRE and fluoroquinolone resistance indicate that longer-term effects on microbial ecology may already be emerging.

In our study, we incorporated effect size measures, including absolute differences and OR, with 95% CI, to provide a more informative interpretation of the results beyond *P*-values or *P* for trend alone (Tables S5–S9). This approach is particularly important given the large sample sizes in our dataset, where small differences in resistance proportions may reach statistical significance but have limited clinical relevance.

Although several comparisons were statistically significant, many absolute differences were modest (e.g. ESBL-producing *E. coli*, +2.4%), underscoring the importance of evaluating effect sizes alongside *P*-values. In contrast, some changes were both statistically and clinically meaningful. For example, resistance to ceftazidime (CAZ) in *E. coli* increased substantially (+19.3%), with OR = 0.423 (95% CI 0.391–0.459). Similarly, the observed decreases in resistance among *A. baumannii* across multiple antibiotics were of notable magnitude and likely reflect clinically relevant changes.

In this study, the start of the COVID-19 pandemic in Mexico was defined as 24 March 2020 (Phase 2, onset of local transmission), following the first confirmed imported case on 27 February 2020,.^33^ The pandemic period ended on 9 May 2023, when the national health emergency was officially lifted.^34^

These cut-offs may not fully reflect country-specific epidemiological dynamics or changes in antibiotic use. We also acknowledge temporal imbalance between periods, which may affect statistical power and comparability; to address this, we complemented between-period comparisons with trend analyses across the full study timeline, which are less sensitive to unequal interval lengths.

Strengths of this study include the participation of multiple centres across Mexico: a large, curated dataset and stratification by organism, specimen and period. However, several limitations should be acknowledged, including the use of commercial broth microdilution systems which may introduce challenges when comparing data across time periods, as variability between panel versions, the potential use of outdated panels and factors such as product recalls could influence susceptibility results. Additional limitations include a lack of molecular resistance data and potential differences in sampling intensity across periods due to pandemic-related disruptions. Furthermore, the lack of stratification of isolates into community-associated versus healthcare-associated settings limits epidemiological interpretation, and participating centres differ substantially in size, healthcare setting and laboratory practices; therefore, the findings should be interpreted with caution, as they may partly reflect structural differences between centres rather than true temporal trends. We also did not analyse the clonal distribution of isolates; therefore, observed changes in resistance may be partly attributable to clonal replacement. Finally, despite standardized procedures across participating centres, variability related to internal quality control, pre-analytical factors (e.g. specimen collection and handling) and inherent limitations of susceptibility testing panels or cards may have influenced the results. Therefore, some observed changes in resistance rates may reflect methodological factors rather than true epidemiological shifts. ESBL detection methods varied across centres and were not standardized; some laboratories used phenotypic confirmatory tests, whereas others relied on automated platform algorithms. This methodological heterogeneity may have introduced systematic bias in the estimated ESBL prevalence, as different platforms and algorithms exhibit variable sensitivity and specificity for ESBL detection.

### Conclusions

This 6 year multicentre surveillance effort reveals important shifts in AMR in Mexico during the COVID-19 era, with increasing ESBL rates, rising VRE prevalence and a concerning upward trend in levofloxacin resistance among *P. aeruginosa* recovered from respiratory specimens. In contrast, significant post-pandemic improvements were observed in carbapenem-resistant *A. baumannii*. These findings emphasize the need for strengthened antimicrobial stewardship to prevent further dissemination of high-risk resistant pathogens in the post-pandemic period.

## Supplementary Material

dlag129_Supplementary_Data

## Supplementary data

Tables S1 to S9 are available as Supplementary data at *JAC-AMR* Online.
