# Supplementary material for: Shift of antibiotic resistance after the COVID-19 pandemic: results from the INVIFAR Network
Source: JAC Antimicrob Resist. 2026 Jul 14;8(4):dlag129. doi: 10.1093/jacamr/dlag129 (PMC13366540; doi:10.1093/jacamr/dlag129)
Supplement: dlag129_Supplementary_Data [file dlag129_supplementary_data.docx]

**Suppl Table 1. Characteristics of participating centers.**

| **Center** | **Pu/Pr** | **Type** | **Level attention** | **Total beds** | **ICU beds** |
| --- | --- | --- | --- | --- | --- |
| Hospital Civil Fray Antonio Alcalde | Pu | Gen | 3rd | 747 | 100 |
| Hospital General León | Pu | Gen | 2nd | 513 | 18 |
| Hospital General Dr. Agustin O'Horan | Pu | Gen | 2nd | 300 | 15 |
| Hospital General Morelia | Pu | Spe | 2nd | 250 | 16 |
| Instituto Nacional de Rehabilitación Luis Guillermo Ibarra Ibarra | Pu | Spe | 3rd | 245 | 18 |
| Instituto Nacional de Ciencias Médicas y Nutrición Salvador Zubirán | Pu | Spe | 3rd | 212 | 22 |
| Hospital Infantil de Morelia | Pu | Ped | 2nd | 209 | 32 |
| Instituto Nacional de Cardiología Ignacio Chávez | Pu | Spe | 3rd | 200 | 30 |
| Hospital Regional "Alta Especialidad" ISSSTE Monterrey | Pu | Spe | 3rd | 200 | 20 |
| Hospital Regional de Alta Especialidad Bicentenario de la Independencia | Pu | Spe | 3rd | 196 | 24 |
| Hospital Regional de Alta Especialidad del Bajío | Pu | Spe | 3rd | 184 | 29 |
| Hospital General de Zona No.1 IMSS Nueva Frontera | Pu | Gen | 2nd | 180 | 28 |
| Hospital para el Niño de Toluca IMIEM | Pu | Ped | 3rd | 180 | 8 |
| Instituto Nacional de Enfermedades Respiratorias Ismael Cosio Villegas | Pu | Spe | 3rd | 175 | 15 |
| Hospital para la Niñez Poblana | Pu | Ped | 3rd | 169 | 15 |
| Hospital General de Querétaro | Pu | Gen | 2nd | 169 | 9 |
| Hospital General del Estado | Pu | Spe | 3rd | 168 | 10 |
| Hospital General Juan María de Salvatierra | Pu | Spe | 2nd | 157 | 18 |
| Hospital Dr. Jesús Gilberto Gómez Maza | Pu | Gen | 2nd | 120 | 12 |
| Hospital General Silao | Pu | Gen | 2nd | 118 | 12 |
| Hospital Regional Universitario IMSS Bienestar | Pu | Gen | 3rd | 108 | 4 |
| Hospital General ISSSTE SLP | Pu | Gen | 2nd | 107 | 4 |
| Hospital General Tapachula | Pu | Gen | 2nd | 92 | 3 |
| Hospital de Especialidades Pediátricas IMSS-Bienestar | Pu | Ped | 3rd | 90 | 19 |
| Hospital Galenia | Pr | Spe | 3rd | 87 | 9 |
| Hospital General de Zona N. 21 IMSS Tepatitlán de Morelos. | Pu | Gen | 2nd | 73 | 13 |
| Hospital Del Niño Morelense | Pu | Spe | 3rd | 67 | 18 |
| Hospital Ángeles Morelia | Pr | Spe | 3rd | 67 | 12 |
| Hospital Regional de Alta Especialidad de Oaxaca | Pu | Spe | 3rd | 66 | 10 |
| Christus Muguerza Hospital Faro del Mayab | Pr | Spe | 3rd | 46 | 6 |
| Hospital del Niño "Dr. Federico Gómez Santos " | Pu | Spe | 3rd | 44 | 13 |
| Hospital Español Veracruz | Pr | Gen | 2nd | 40 | 6 |
| Hospital de la Madre y el Niño Guerrerense | Pu | O&G | 2nd | 37 | 0 |
| Hospital Adolfo López Mateos | Pu | Gen | 2nd | 33 | 2 |
| Universidad Autónoma de Guerrero | Pr | NHBL | NA | NA | NA |
| Departamento de Microbiología, Facultad de Medicina, Universidad Autónoma de Nuevo León | Pu | NHBL | NA | NA | NA |
| Lapi Laboratorio Médico | Pr | NHBL | NA | NA | NA |
| Laboratorio Estatal de Salud Pública | Pu | NHBL | NA | NA | NA |
| Universidad de Sonora | Pu | NHBL | NA | NA | NA |
| Laboratorios Clínicos de Referencia MICROTEC | Pr | NHBL | NA | NA | NA |
| Laboratorios Preciado Montes | Pr | NHBL | NA | NA | NA |
| Universidad de Sonora | Pu | NHBL | NA | NA | NA |
| Laboratorio Dorado | Pr | NHBL | NA | NA | NA |
| BioDiagnostics Laboratorio Clínico | Pr | NHBL | NA | NA | NA |
| Centro Universitario de Salud, Universidad Autónoma de Nuevo León | Pu | NHBL | NA | NA | NA |
| Universidad Autónoma de Baja California, Facultad de Medicina | Pu | NHBL | NA | NA | NA |
| Centro Integral de Atención a la Salud Unidad Sur ISSSTESON | Pu | NHBL | NA | NA | NA |

Pu: public, Pr: private, NHBL: non-hospital-based laboratory, Spe: specialties, Ped: pediatric, Gen: general, O&G: obstetrics and gynecology, NA: Not apply,

**Suppl Table 2. Distribution of resistant, intermediate, and susceptible isolates by clinical specimen.**

|  | **Blood** | | | | **Urine** | | | | **LRT** | | | | **Abscess and biopsies** | | | | **CSF** | | | |  |
| --- | --- | --- | --- | --- | --- | --- | --- | --- | --- | --- | --- | --- | --- | --- | --- | --- | --- | --- | --- | --- | --- |
|  | **n** | **%R** | **%I** | **%S** | **n** | **%R** | **%I** | **%S** | **n** | **%R** | **%I** | **%S** | **n** | **%R** | **%I** | **%S** | **n** | **%R** | **%I** | **%S** | ***p*** |
| ***E. coli*** | | | | | | | | | | | | | | | | | | | | | |
| ESBL | 2,555 | 60.4* |  |  | 32,175 | 46.3* |  |  | 1,284 | 68.0 |  |  | 3,653 | 62.9* |  |  | 75 | 77.3 |  |  | **<0.001** |
| SAM | 4,558 | 46.0 | 19.6 |  | 36,735 | 38.7 | 19.5 | 41.8 | 1,635 | 49.7 | 16.9 | 33.5 | 5,104 | 47.0 | 16.7 | 36.3 | 91 | 47.3 | 19.8 | 33.0 | **<0.001** |
| CRO | 4,149 | 62.1 | 0.1 | 37.8 | 43,873 | 45.4 | 0.2 | 54.5 | 1,697 | 75.8 | 0.2 | 24.0 | 5,208 | 66.7 | 0.1 | 33.2 | 93 | 76.3 | 0.0 | 23.7 | **<0.001** |
| ETP | 4,549 | 7.0 | 0.7 | 92.3 | 43,735 | 1.8 | 0.4 | 97.8 | 1,742 | 7.1 | 1.0 | 91.9 | 5,222 | 6.2 | 0.4 | 93.4 | 92 | 3.3 | 1.1 | 95.7 | **<0.001** |
| IPM | 3,159 | 6.6 | 0.6 | 92.9 | 20,312 | 1.2 | 0.6 | 98.2 | 1,332 | 4.4 | 0.7 | 94.9 | 3,916 | 5.1 | 0.9 | 94.1 | 63 | 0.0 | 0.0 | 100.0 | **<0.001** |
| MEM | 4,289 | 5.9 | 0.3 | 93.8 | 42,504 | 1.4 | 0.2 | 98.5 | 1,725 | 6.6 | 0.5 | 92.9 | 4,981 | 5.2 | 0.3 | 94.5 | 94 | 2.1 | 0.0 | 97.9 | **<0.001** |
| SXT | 1,850 | 62.2 | 0.0 | 37.8 | 38,355 | 53.7 | 0.0 | 46.3 | 968 | 62.1 | 0.1 | 37.8 | 2,413 | 65.0 | 0.2 | 34.9 | 43 | 65.1 | 0.0 | 34.9 | **<0.001** |
| ***K. pneumoniae*** | | | | | | | | | | | | | | | | | | | | | |
| ESBL | 1,825 | 51.8* |  |  | 5,415 | 43.7* |  |  | 1,991 | 45.7* |  |  | 1,538 | 57.5* |  |  | 63 | 73.0* |  |  | **<0.001** |
| SAM | 2,501 | 50.1 | 6.1 | 43.8 | 6,671 | 40.5 | 7.8 | 51.6 | 2,544 | 44.7 | 7.2 | 48.1 | 1,968 | 51.0 | 7.4 | 41.6 | 78 | 67.9 | 2.6 | 29.5 | **<0.001** |
| CRO | 2,357 | 56.0 | 0.2 | 43.9 | 7,522 | 42.5 | 0.2 | 57.3 | 2,560 | 50.2 | 0.2 | 49.6 | 1,964 | 56.3 | 0.2 | 43.6 | 71 | 77.5 | 0.0 | 22.5 | **<0.001** |
| ETP | 2,484 | 11.6 | 0.4 | 88.0 | 7,364 | 4.0 | 0.8 | 95.2 | 2,603 | 8.7 | 0.6 | 90.7 | 1,920 | 7.2 | 0.3 | 92.5 | 75 | 16.0 | 0.0 | 84.0 | **<0.001** |
| IPM | 1,749 | 10.9 | 1.5 | 87.7 | 3,198 | 3.9 | 0.9 | 95.1 | 1,986 | 7.4 | 0.7 | 92.0 | 1,541 | 6.5 | 1.9 | 91.6 | 57 | 21.1 | 0.0 | 78.9 | **<0.001** |
| MEM | 2,432 | 10.2 | 0.7 | 89.1 | 7,159 | 3.1 | 0.3 | 96.6 | 2,572 | 7.5 | 0.4 | 92.1 | 1,862 | 5.9 | 0.5 | 93.6 | 78 | 14.1 | 0.0 | 85.9 | **<0.001** |
| ***E. cloacae*** | | | | | | | | | | | | | | | | | | | | | |
| ETP | 1,186 | 9.1 | 1.9 | 89.0 | 1,065 | 15.9 | 2.7 | 81.4 | 1,255 | 9.6 | 5.7 | 84.6 | 1,124 | 7.2 | 2.2 | 90.6 | ND | ND | ND | ND | **<0.001** |
| IPM | 804 | 8.2 | 8.0 | 83.8 | 851 | 10.1 | 7.8 | 82.1 | 1,090 | 4.9 | 5.1 | 90.0 | 1,006 | 2.4 | 1.9 | 95.7 | ND | ND | ND | ND | **<0.001** |
| MEM | 1,112 | 6.5 | 1.2 | 92.4 | 1,015 | 11.2 | 0.8 | 88.0 | 1,240 | 4.0 | 0.4 | 95.6 | 1,106 | 2.8 | 0.2 | 97.0 | ND | ND | ND | ND | **<0.001** |
| SXT | 584 | 22.1 | 0.2 | 77.7 | 1,246 | 38.9 | 0.0 | 61.1 | 844 | 13.3 | 0.0 | 86.7 | 678 | 18.6 | 0.1 | 81.3 | ND | ND | ND | ND | **<0.001** |
| ***A. baumannii*** | | | | | | | | | | | | | | | | | | | | | |
| SAM | 1,121 | 54.8 | 15.7 | 29.5 | 389 | 61.2 | 14.1 | 24.7 | 1,562 | 57.0 | 19.1 | 23.9 | 784 | 62.5 | 13.6 | 23.9 | 56 | 62.5 | 12.5 | 25.0 | **0.012** |
| TZP | 926 | 81.9 | 1.0 | 17.2 | 280 | 91.4 | 0.7 | 7.9 | 1,441 | 83.9 | 0.7 | 15.4 | 702 | 84.6 | 1.1 | 14.2 | 56 | 80.4 | 0.0 | 19.6 | **0.003** |
| FEP | 1,146 | 63.8 | 11.2 | 25.0 | 432 | 70.6 | 10.6 | 18.8 | 1,671 | 71.7 | 8.1 | 20.2 | 809 | 68.0 | 15.6 | 16.4 | 61 | 65.6 | 11.5 | 23.0 | **<0.001** |
| IPM | 824 | 78.9 | 0.0 | 21.1 | 237 | 84.8 | 0.8 | 14.3 | 1,269 | 82.0 | 0.6 | 17.4 | 696 | 78.9 | 1.1 | 20.0 | 48 | 77.1 | 0.0 | 22.9 | 0.075 |
| MEM | 1,175 | 72.7 | 0.6 | 26.7 | 441 | 78.2 | 1.6 | 20.2 | 1,699 | 79.3 | 0.9 | 19.7 | 820 | 80.9 | 1.1 | 18.0 | 62 | 77.4 | 0.0 | 22.6 | **<0.001** |
| GEN | 1,048 | 54.7 | 5.7 | 39.6 | 413 | 62.5 | 5.3 | 32.2 | 1,563 | 58.9 | 7.1 | 34.0 | 730 | 67.3 | 8.2 | 24.5 | 60 | 61.7 | 6.7 | 31.7 | **<0.001** |
| CIP | 1,147 | 74.0 | 0.1 | 25.9 | 460 | 84.1 | 0.2 | 15.7 | 1,647 | 80.6 | 0.2 | 19.2 | 805 | 84.0 | 0.2 | 15.8 | 63 | 77.8 | 0.0 | 22.2 | **<0.001** |
| ***P. aeruginosa*** | | | | | | | | | | | | | | | | | | | | | |
| CZT | 318 | 39.9 | 4.1 | 56.0 | 341 | 28.4 | 4.1 | 67.4 | 244 | 15.2 | 12.3 | 72.5 | 526 | 24.0 | 4.0 | 72.1 | ND | ND | ND | ND | **<0.001** |
| CZA | 464 | 41.4 | 0.6 | 58.0 | 664 | 32.5 | 0.3 | 67.2 | 255 | 23.1 | 0.0 | 76.9 | 840 | 33.6 | 0.5 | 66.0 | ND | ND | ND | ND | **<0.001** |
| TZP | 322 | 29.2 | 9.5 | 61.3 | 2,835 | 22.6 | 8.1 | 69.3 | 2,839 | 30.5 | 9.1 | 60.4 | 2,291 | 28.4 | 13.5 | 58.1 | ND | ND | ND | ND | **<0.001** |
| CAZ | 698 | 39.6 | 6.4 | 54.0 | 1,285 | 27.3 | 2.6 | 70.1 | 928 | 32.4 | 4.7 | 62.8 | 1,525 | 40.2 | 6.8 | 53.0 | ND | ND | ND | ND | **<0.001** |
| FEP | 180 | 25.6 | 7.3 | 67.1 | 3,933 | 32.0 | 5.5 | 62.4 | 3,431 | 24.0 | 9.3 | 66.6 | 2,602 | 27.9 | 10.7 | 61.3 | ND | ND | ND | ND | **<0.001** |
| IPM | 2,573 | 43.2 | 2.5 | 54.2 | 2,511 | 45.1 | 3.1 | 51.7 | 2,668 | 46.1 | 2.5 | 51.4 | 2,366 | 50.5 | 0.8 | 48.7 | ND | ND | ND | ND | **<0.001** |
| MEM | 2,103 | 36.0 | 5.1 | 59.0 | 4,168 | 36.0 | 4.2 | 59.8 | 3,599 | 38.8 | 6.9 | 54.2 | 2,774 | 42.4 | 7.2 | 50.4 | ND | ND | ND | ND | **<0.001** |
| ***S. aureus*** | | | | | | | | | | | | | | | | | | | | | |
| OXA | 2,942 | 23.4 | 0.0 | 76.6 | 703 | 19.1 | 0.0 | 80.9 | 2,329 | 17.0 | 0.0 | 83.0 | 3,478 | 25.8 | 0.0 | 74.2 | 113 | 21.2 | 0.0 | 78.8 | **<0.001** |
| GEN | 2,273 | 10.1 | 4.2 | 85.7 | 751 | 24.0 | 5.3 | 70.7 | 1,920 | 7.1 | 2.1 | 90.8 | 2,471 | 7.1 | 4.2 | 88.7 | 79 | 11.4 | 3.8 | 84.8 | **<0.001** |
| CIP | 2,923 | 24.4 | 1.8 | 73.8 | 698 | 22.2 | 1.7 | 76.1 | 2,311 | 18.3 | 1.2 | 80.5 | 3,456 | 27.8 | 2.3 | 69.8 | 112 | 17.0 | 1.8 | 81.3 | **<0.001** |
| LVX | 2,631 | 25.1 | 0.7 | 74.2 | 571 | 25.2 | 2.5 | 72.3 | 1,497 | 21.1 | 0.5 | 78.4 | 3,322 | 28.5 | 0.6 | 71.0 | 96 | 16.7 | 1.0 | 82.3 | **<0.001** |
| SXT | 2,871 | 4.5 | 0.0 | 95.5 | 890 | 12.9 | 0.0 | 87.1 | 2,195 | 4.3 | 0.0 | 95.7 | 3,417 | 6.4 | 0.0 | 93.6 | 110 | 6.4 | 0.0 | 93.6 | **<0.001** |
| CLI | 2,812 | 34.3 | 0.5 | 65.2 | 509 | 30.1 | 1.0 | 69.0 | 2,266 | 27.2 | 0.4 | 72.5 | 3,300 | 42.0 | 0.0 | 57.9 | 110 | 30.0 | 0.0 | 70.0 | **<0.001** |
| ERY | 2,771 | 35.0 | 1.3 | 63.7 | 505 | 31.3 | 1.8 | 66.9 | 2,251 | 26.6 | 0.7 | 72.7 | 3,286 | 39.8 | 0.8 | 59.4 | 110 | 29.1 | 0.9 | 70.0 | **<0.001** |
| LNZ | 2,814 | 2.4 | 0.0 | 97.6 | 667 | 2.7 | 0.0 | 97.3 | 2,294 | 1.7 | 0.0 | 98.3 | 3,337 | 0.3 | 0.0 | 99.7 | 112 | 0.0 | 0.0 | 100.0 | **<0.001** |
| ***E. faecium*** | | | | | | | | | | | | | | | | | | | | | |
| PEN | 167 | 71.9 | 0.0 | 28.1 | 466 | 85.2 | 0.0 | 14.8 | ND | ND | ND | ND | 342 | 57.9 | 0.3 | 41.8 | ND | ND | ND | ND | **<0.001** |
| AMP | 492 | 69.7 | 0.0 | 30.3 | 1,181 | 84.5 | 0.0 | 15.5 | ND | ND | ND | ND | 846 | 57.7 | 0.0 | 42.3 | ND | ND | ND | ND | **<0.001** |
| CIP | 486 | 56.8 | 16.3 | 27.0 | 1,168 | 63.6 | 15.3 | 21.1 | ND | ND | ND | ND | 869 | 38.7 | 21.7 | 39.6 | ND | ND | ND | ND | **<0.001** |
| LVX | 441 | 53.1 | 11.6 | 35.4 | 1,059 | 61.4 | 7.1 | 31.5 | ND | ND | ND | ND | 847 | 36.5 | 10.0 | 53.5 | ND | ND | ND | ND | **<0.001** |
| ERY | 469 | 80.2 | 13.9 | 6.0 | 1,057 | 88.1 | 8.3 | 3.6 | ND | ND | ND | ND | 871 | 63.5 | 27.9 | 8.6 | ND | ND | ND | ND | **<0.001** |
| LNZ | 476 | 0.2 | 2.9 | 96.8 | 1,141 | 3.9 | 3.2 | 92.9 | ND | ND | ND | ND | 834 | 0.7 | 1.0 | 98.3 | ND | ND | ND | ND | **<0.001** |
| VAN | 476 | 36.6 | 0.0 | 63.4 | 1,161 | 34.5 | 0.5 | 65.0 | ND | ND | ND | ND | 855 | 19.5 | 0.1 | 80.4 | ND | ND | ND | ND | **<0.001** |

* Percent positive

AMP: Ampicillin, CAZ: Ceftazidime, CIP: Ciprofloxacin, CLI: Clindamycin, CRO: Ceftriaxone, CZA: Ceftazidime/Avibactam, CZT: Ceftolozane/Tazobactam, ERY: Erythromycin, ESBL: extended spectrum beta-lactamase, ETP: Ertapenem, FEP: Cefepime, GEN: Gentamicin, IPM: Imipenem, LNZ: Linezolid, LVX: Levofloxacin, MEM: Meropenem, OXA: Oxacillin, PEN: Penicillin, SAM: Ampicillin/Sulbactam, SXT: Sulfamethoxazole/Trimethoprim, TZP: Piperacillin/Tazobactam, VAN: Vancomycin, Not Determined.

**Suppl Table 3. Distribution of resistant, intermediate, and susceptible isolates by period.**

|  | **Pre-pandemic** | | | | **Pandemic** | | | | **Post-pandemic** | | | |  |  |
| --- | --- | --- | --- | --- | --- | --- | --- | --- | --- | --- | --- | --- | --- | --- |
|  | **n** | **%R** | **%I** | **%S** | **n** | **%R** | **%I** | **%S** | **n** | **%R** | **%I** | **%S** | ***p value*** | ***p for trend*** |
| ***E. coli*** | | | | | | | | | | | | | | |
| ESBL | 3,924 | 46.5 |  |  | 7,432 | 47.8* |  |  | 13,299 | 48.9* |  |  | **0.018** | **0.005** |
| AMP | 5,236 | 75.1 | 1.2 | 23.7 | 10,192 | 77.2 | 1.0 | 21.8 | 5,052 | 75.9 | 1.1 | 23.1 | **0.016** | 0.411 |
| AMC | 1,230 | 16.3 | 22.4 | 61.3 | 1,074 | 11.6 | 20.9 | 67.4 | 1,588 | 14.2 | 20.5 | 65.3 | **0.003** | 0.100 |
| SAM | 5,602 | 41.3 | 22.0 | 36.7 | 13,361 | 41.3 | 18.1 | 40.6 | 14,804 | 39.7 | 17.9 | 42.3 | **<0.001** | **<0.001** |
| CAZ | 5,263 | 44.7 | 0.2 | 55.1 | 6,923 | 50.1 | 0.2 | 49.7 | 5,090 | 64.0 | 2.6 | 33.4 | **<0.001** | **<0.001** |
| CRO | 7,009 | 47.2 | 0.2 | 52.6 | 13,514 | 51.1 | 0.1 | 48.8 | 14,703 | 48.8 | 0.1 | 51.1 | **<0.001** | 0.356 |
| CTX | 3,032 | 40.4 | 0.4 | 59.2 | 6,632 | 45.2 | 0.2 | 54.6 | 3,209 | 45.3 | 0.7 | 54.0 | **<0.001** | **<0.001** |
| FEP | 6,907 | 34.1 | 2.2 | 63.7 | 14,440 | 37.1 | 4.2 | 58.7 | 14,554 | 38.4 | 5.9 | 55.7 | **<0.001** | **<0.001** |
| FOX | 3,134 | 13.1 | 5.5 | 81.4 | 5,171 | 14.5 | 5.1 | 80.4 | 3,860 | 19.7 | 6.8 | 73.5 | **<0.001** | **<0.001** |
| ETP | 7,818 | 2.0 | 0.3 | 97.7 | 14,394 | 2.7 | 0.2 | 97.1 | 14,461 | 3.2 | 0.4 | 96.4 | **<0.001** | **<0.001** |
| IPM | 3,484 | 2.1 | 0.5 | 97.4 | 6,706 | 3.4 | 0.4 | 96.1 | 6,615 | 2.3 | 0.9 | 96.7 | **<0.001** | 0.912 |
| MEM | 7,518 | 1.2 | 0.1 | 98.7 | 14,532 | 2.6 | 0.2 | 97.3 | 13,048 | 2.7 | 0.2 | 97.1 | **<0.001** | **<0.001** |
| CIP | 7,040 | 61.3 | 3.9 | 34.8 | 14,642 | 62.3 | 6.1 | 31.6 | 16,275 | 58.1 | 16.3 | 25.6 | **<0.001** | **<0.001** |
| SXT | 5,232 | 52.9 | 0.0 | 47.0 | 9,804 | 55.6 | 0.0 | 44.3 | 12,628 | 56.3 | 0.0 | 43.7 | **<0.001** | **<0.001** |
| ***K. pneumoniae*** | | | | | | | | | | | | | | |
| ESBL | 1,066 | 45.9* |  |  | 2,254 | 44.1* |  |  | 2,785 | 44.0* |  |  | 0.548 | 0.376 |
| SAM | 1,455 | 44.5 | 6.5 | 48.9 | 3,501 | 44.0 | 6.5 | 49.5 | 3,794 | 38.2 | 7.3 | 54.5 | **<0.001** | **<0.001** |
| CAZ | 1,146 | 37.8 | 0.0 | 62.2 | 1,773 | 40.5 | 0.6 | 58.9 | 1,317 | 54.4 | 1.4 | 44.2 | **<0.001** | **<0.001** |
| CRO | 1,655 | 42.1 | 0.1 | 57.9 | 3,683 | 45.9 | 0.2 | 53.9 | 3,620 | 43.6 | 0.1 | 56.4 | **0.015** | 0.747 |
| FEP | 1,711 | 28.5 | 1.3 | 70.1 | 3,688 | 27.7 | 2.7 | 69.6 | 3,387 | 36.3 | 3.7 | 60.0 | **<0.001** | **<0.001** |
| ETP | 1,842 | 3.8 | 0.2 | 96.0 | 3,678 | 6.9 | 0.1 | 93.0 | 3,380 | 4.1 | 0.5 | 95.5 | **<0.001** | 0.428 |
| IPM | 1,021 | 3.6 | 0.4 | 96.0 | 2,215 | 7.0 | 0.7 | 92.3 | 1,471 | 4.5 | 1.4 | 94.1 | **<0.001** | 0.659 |
| MEM | 1,755 | 3.6 | 0.1 | 96.3 | 3,711 | 5.7 | 0.3 | 94.0 | 3,056 | 3.4 | 0.4 | 96.2 | **<0.001** | 0.221 |
| CIP | 1,676 | 37.4 | 5.5 | 57.0 | 3,676 | 42.4 | 5.4 | 52.2 | 3,993 | 37.7 | 10.2 | 52.1 | **0.001** | 0.483 |
| SXT | 1,078 | 43.9 | 0.0 | 56.1 | 2,052 | 48.0 | 0.0 | 52.0 | 2,452 | 48.4 | 0.1 | 51.5 | **0.035** | **0.024** |
| ***E. cloacae*** | | | | | | | | | | | | | | |
| FEP | 432 | 5.1 | 2.3 | 92.6 | 1,348 | 4.5 | 4.5 | 91.1 | 1,146 | 10.1 | 3.6 | 86.3 | **<0.001** | **<0.001** |
| ETP | 454 | 4.4 | 3.5 | 92.1 | 1,347 | 7.4 | 3.3 | 89.2 | 1,152 | 9.3 | 3.5 | 87.2 | **0.004** | **0.001** |
| IPM | 374 | 2.9 | 2.4 | 94.7 | 1,084 | 1.7 | 2.8 | 95.6 | 821 | 3.5 | 4.3 | 92.2 | **0.028** | 0.186 |
| MEM | 462 | 2.4 | 0.0 | 97.6 | 1,353 | 2.3 | 0.9 | 96.8 | 961 | 5.2 | 0.7 | 94.1 | **<0.001** | **0.001** |
| CIP | 462 | 7.6 | 2.2 | 90.3 | 1,325 | 9.4 | 2.9 | 87.8 | 1,160 | 15.4 | 5.7 | 78.9 | **<0.001** | **<0.001** |
| SXT | 292 | 7.5 | 0.0 | 92.5 | 707 | 12.6 | 0.0 | 87.4 | 932 | 35.5 | 0.0 | 64.5 | **<0.001** | **<0.001** |
| ***A. baumannii*** | | | | | | | | | | | | | | |
| SAM | 442 | 71.5 | 10.0 | 18.6 | 1,084 | 59.5 | 18.2 | 22.3 | 653 | 40.7 | 21.7 | 37.5 | **<0.001** | **<0.001** |
| TZP | 457 | 86.2 | 0.9 | 12.9 | 915 | 83.9 | 0.9 | 15.2 | 422 | 69.7 | 1.7 | 28.7 | **<0.001** | **<0.001** |
| FEP | 552 | 82.2 | 0.5 | 17.2 | 1,131 | 79.4 | 1.4 | 19.2 | 691 | 48.5 | 20.3 | 31.3 | **<0.001** | **<0.001** |
| IPM | 298 | 77.2 | 0.3 | 22.5 | 860 | 82.3 | 0.1 | 17.6 | 374 | 63.9 | 0.8 | 35.3 | **<0.001** | **<0.001** |
| MEM | 550 | 82.5 | 0.5 | 16.9 | 1,132 | 78.6 | 0.3 | 21.1 | 700 | 65.1 | 1.1 | 33.7 | **<0.001** | **<0.001** |
| AMK | 131 | 51.9 | 0.0 | 48.1 | 267 | 53.9 | 3.4 | 42.7 | 379 | 38.5 | Ab | 54.6 | **0.001** | **0.004** |
| GEN | 457 | 72.9 | 6.6 | 20.6 | 1,087 | 60.5 | 5.1 | 34.4 | 620 | 46.5 | 11.9 | 41.6 | **<0.001** | **<0.001** |
| CIP | 559 | 86.8 | 0.0 | 13.2 | 1,121 | 80.2 | 0.2 | 19.6 | 692 | 67.9 | 0.1 | 31.9 | **<0.001** | **<0.001** |
| LVX | 65 | 52.3 | 0.0 | 47.7 | 136 | 74.3 | 0.7 | 25.0 | 49 | 55.1 | 0.0 | 44.9 | **0.002** | 0.501 |
| SXT | 223 | 80.3 | 0.4 | 19.3 | 373 | 67.3 | 0.0 | 32.7 | 337 | 63.2 | 0.0 | 36.8 | **<0.001** | **<0.001** |
| ***P. aeruginosa*** | | | | | | | | | | | | | | |
| TZP | 1,556 | 21.1 | 7.1 | 71.8 | 2,601 | 23.5 | 8.6 | 67.9 | 2,200 | 21.4 | 6.6 | 72.0 | 0.037 | 0.897 |
| CAZ | 969 | 18.8 | 4.1 | 77.1 | 1,525 | 24.4 | 3.8 | 71.8 | 1,138 | 20.0 | 1.5 | 78.5 | **0.001** | 0.860 |
| FEP | 1,626 | 18.2 | 6.2 | 75.6 | 3,675 | 19.1 | 6.7 | 74.2 | 2,955 | 15.6 | 8.6 | 75.8 | **0.003** | **0.017** |
| IPM | 1,223 | 35.8 | 0.8 | 63.4 | 2,383 | 36.7 | 2.1 | 61.2 | 2,060 | 32.4 | 3.3 | 64.4 | **0.022** | 0.062 |
| MEM | 1,685 | 29.9 | 5.6 | 64.5 | 3,748 | 30.1 | 5.5 | 64.4 | 3,457 | 25.8 | 7.8 | 66.4 | **0.001** | **0.002** |
| CIP | 1,820 | 28.1 | 5.1 | 66.8 | 3,682 | 24.1 | 5.2 | 70.7 | 3,437 | 26.0 | 4.6 | 69.4 | **0.005** | 0.648 |
| LVX | 353 | 18.1 | 8.8 | 73.1 | 718 | 26.6 | 8.2 | 65.2 | 348 | 30.2 | 7.5 | 62.4 | **0.001** | **<0.001** |
| ***E. faecium*** | | | | | | | | | | | | | | |
| AMP | 398 | 66.6 | 0.0 | 33.4 | 664 | 73.0 | 0.0 | 27.0 | 686 | 73.6 | 0.0 | 26.4 | **0.031** | **0.022** |
| CIP | 373 | 56.0 | 15.0 | 29.0 | 648 | 49.8 | 19.9 | 30.2 | 649 | 54.7 | 16.8 | 28.5 | 0.405 | 0.870 |
| LVX | 303 | 55.8 | 7.3 | 37.0 | 578 | 46.5 | 9.7 | 43.8 | 608 | 54.3 | 8.4 | 37.3 | **0.015** | 0.711 |
| ERY | 332 | 78.0 | 18.1 | 3.9 | 622 | 83.0 | 12.5 | 4.5 | 589 | 74.9 | 17.3 | 7.8 | **0.008** | **0.006** |
| LNZ | 367 | 0.3 | 0.5 | 99.2 | 656 | 1.1 | 0.8 | 98.2 | 607 | 0.7 | 0.7 | 98.7 | 0.346 | 0.637 |
| VAN | 388 | 29.1 | 0.0 | 70.9 | 657 | 19.8 | 0.3 | 79.9 | 610 | 43.1 | 0.0 | 56.9 | **<0.001** | **<0.001** |
| ***S. aureus*** | | | | | | | | | | | | | | |
| OXA | 1,427 | 13.9 | 0.0 | 86.1 | 3,422 | 19.0 | 0.0 | 81.0 | 3,549 | 16.5 | 0.0 | 83.5 | **<0.001** | 0.315 |
| FOX | 246 | 10.2 | 0.0 | 89.8 | 531 | 13.0 | 0.0 | 87.0 | 239 | 5.0 | 0.0 | 95.0 | **0.004** | **0.068** |
| GEN | 1,526 | 9.5 | 3.6 | 86.9 | 3,462 | 9.8 | 4.2 | 86.0 | 1,362 | 16.4 | 3.8 | 79.8 | **<0.001** | **<0.001** |
| CIP | 1,569 | 16.1 | 1.8 | 82.1 | 3,700 | 19.5 | 2.8 | 77.6 | 3,604 | 18.4 | 1.6 | 80.0 | **0.007** | 0.232 |
| LVX | 1,185 | 15.9 | 0.3 | 83.7 | 3,098 | 18.8 | 1.9 | 79.3 | 3,248 | 19.2 | 0.3 | 80.5 | **0.035** | 0.273 |
| SXT | 1,493 | 5.1 | 0.0 | 94.9 | 3,504 | 4.9 | 0.2 | 94.9 | 3,016 | 6.4 | 0.0 | 93.6 | **0.025** | **0.028** |
| CLI | 1,503 | 27.8 | 0.4 | 71.8 | 3,581 | 31.1 | 1.4 | 67.5 | 2,764 | 31.1 | 0.4 | 68.6 | **0.032** | 0.061 |
| ERY | 1,417 | 29.7 | 0.9 | 69.4 | 3,361 | 31.7 | 1.2 | 67.1 | 2,753 | 30.8 | 1.8 | 67.4 | 0.355 | 0.520 |
| LNZ | 1,425 | 0.0 | 0.0 | 100.0 | 3,442 | 0.5 | 0.0 | 99.5 | 2,865 | 0.5 | 0.0 | 99.5 | **0.026** | **0.026** |

* Percent positive. AMC: Amoxicillin/Clavulanic acid, AMK: Amikacin, AMP: Ampicillin, CAZ: Ceftazidime, CIP: Ciprofloxacin, CLI: Clindamycin, CRO: Ceftriaxone, CTX: Cefotaxime, ERY: Erythromycin, ESBL: extended spectrum beta-lactamase, ETP: Ertapenem, FEP: Cefepime, FOX: Cefoxitin, GEN: Gentamicin, IPM: Imipenem, LNZ: Linezolid, LVX: Levofloxacin, MEM: Meropenem, OXA: Oxacillin, SAM: Ampicillin/Sulbactam, SXT: Sulfamethoxazole/Trimethoprim, TZP: Piperacillin/Tazobactam, VAN: Vancomycin, ND: Not Determined.

**Suppl Table 4A. Distribution of resistant, intermediate, and susceptible isolates by period in blood.**

|  | **Pre-pandemic** | | | | **Pandemic** | | | | **Post-pandemic** | | | |  |  |
| --- | --- | --- | --- | --- | --- | --- | --- | --- | --- | --- | --- | --- | --- | --- |
|  | **n** | **%R** | **%I** | **%S** | **n** | **%R** | **%I** | **%S** | **n** | **%R** | **%I** | **%S** | ***p value*** | ***p for trend*** |
| ***E. coli*** | | | | | | | | | | | | | | |
| ESBL | 117 | 53.8 |  | 46.2 | 402 | 58.2 |  | 41.8 | 410 | 64.1 |  | 35.9 | 0.071 | **0.022** |
| AMP | 186 | 84.9 | 0.0 | 15.1 | 172 | 90.1 | 0.6 | 9.3 | 99 | 87.9 | 0.0 | 12.1 | 0.261 | 0.323 |
| SAM | 545 | 43.3 | 27.9 | 28.8 | 1,089 | 44.6 | 18.2 | 37.2 | 1,083 | 42.6 | 18.2 | 39.2 | **0.029** | **0.010** |
| CAZ | 587 | 51.6 | 0.0 | 48.4 | 757 | 52.2 | 0.0 | 47.8 | 735 | 53.6 | 1.2 | 45.2 | 0.585 | 0.327 |
| CRO | 674 | 53.4 | 0.0 | 46.6 | 1,107 | 56.8 | 0.0 | 43.2 | 557 | 65.4 | 0.0 | 34.6 | **<0.001** | **<0.001** |
| FEP | 566 | 47.9 | 1.2 | 50.9 | 1,099 | 44.9 | 2.6 | 52.5 | 857 | 62.9 | 4.9 | 32.2 | **<0.001** | **<0.001** |
| FOX | 619 | 16.0 | 4.2 | 79.8 | 981 | 16.1 | 3.6 | 80.4 | 133 | 14.3 | 2.3 | 83.5 | 0.830 | 0.702 |
| DOR | 35 | 5.7 | 0.0 | 94.3 | 165 | 3.0 | 1.2 | 95.8 | 32 | 3.1 | 3.1 | 93.8 | 0.738 | 0.564 |
| ETP | 688 | 7.4 | 1.6 | 91.0 | 1,103 | 7.5 | 0.2 | 92.3 | 863 | 10.2 | 0.7 | 89.1 | 0.061 | **0.044** |
| IPM | 610 | 5.7 | 0.7 | 93.6 | 982 | 7.2 | 0.4 | 92.4 | 442 | 14.9 | 0.7 | 84.4 | **<0.001** | **<0.001** |
| MEM | 680 | 5.1 | 0.0 | 94.9 | 1,099 | 7.0 | 0.0 | 93.0 | 620 | 11.8 | 0.8 | 87.4 | **<0.001** | **<0.001** |
| CIP | 673 | 60.6 | 1.9 | 37.4 | 1,103 | 60.8 | 5.2 | 34.0 | 1,098 | 57.9 | 23.7 | 18.4 | **<0.001** | **<0.001** |
| SXT | 185 | 59.5 | 0.0 | 40.5 | 172 | 66.9 | 0.0 | 33.1 | 202 | 60.4 | 0.0 | 39.6 | 0.293 | 0.879 |
| ***K. pneumoniae*** | | | | | | | | | | | | | | |
| ESBL | 86 | 43.0 |  | 57.0 | 333 | 45.3 |  | 54.7 | 218 | 56.4 |  | 43.6 | **0.020** | **0.010** |
| SAM | 203 | 46.3 | 4.4 | 49.3 | 555 | 55.0 | 3.2 | 41.8 | 456 | 41.0 | 9.2 | 49.8 | **0.001** | 0.099 |
| CRO | 227 | 46.3 | 0.0 | 53.7 | 568 | 54.2 | 0.0 | 45.8 | 284 | 54.6 | 0.0 | 45.4 | 0.095 | 0.076 |
| FEP | 218 | 28.9 | 3.2 | 67.9 | 571 | 32.4 | 3.5 | 64.1 | 357 | 44.0 | 3.6 | 52.4 | **<0.001** | **<0.001** |
| ETP | 238 | 10.9 | 0.4 | 88.7 | 567 | 16.2 | 0.0 | 83.8 | 358 | 7.8 | 0.3 | 91.9 | **0.001** | 0.102 |
| IPM | 182 | 9.9 | 0.5 | 89.6 | 446 | 14.6 | 0.7 | 84.8 | 211 | 8.5 | 2.4 | 89.1 | 0.058 | 0.636 |
| MEM | 230 | 10.4 | 0.4 | 89.1 | 566 | 13.4 | 0.7 | 85.9 | 311 | 8.4 | 0.0 | 91.6 | 0.063 | 0.316 |
| CIP | 227 | 34.8 | 7.0 | 58.1 | 566 | 47.0 | 3.0 | 50.0 | 462 | 36.1 | 10.8 | 53.0 | **0.006** | 0.987 |
| SXT | 66 | 43.9 | 0.0 | 56.1 | 134 | 63.4 | 0.0 | 36.6 | 111 | 65.8 | 0.0 | 34.2 | **0.010** | **0.008** |
| ***E. cloacae*** | | | | | | | | | | | | | | |
| FEP | 70 | 1.4 | 2.9 | 95.7 | 226 | 4.0 | 3.5 | 92.5 | 258 | 7.0 | 1.2 | 91.9 | 0.121 | **0.040** |
| ETP | 75 | 2.7 | 4.0 | 93.3 | 222 | 8.6 | 2.3 | 89.2 | 263 | 6.5 | 1.1 | 92.4 | 0.211 | 0.632 |
| IPM | 62 | 1.6 | 0.0 | 98.4 | 179 | 2.2 | 5.0 | 92.7 | 112 | 4.5 | 8.0 | 87.5 | 0.391 | 0.197 |
| MEM | 75 | 1.3 | 0.0 | 98.7 | 222 | 3.2 | 3.6 | 93.2 | 185 | 4.3 | 1.1 | 94.6 | 0.469 | 0.227 |
| CIP | 75 | 8.0 | 1.3 | 90.7 | 221 | 12.2 | 2.3 | 85.5 | 267 | 11.2 | 7.1 | 81.6 | 0.578 | 0.489 |
| ***A. baumannii*** | | | | | | | | | | | | | | |
| SAM | 72 | 70.8 | 5.6 | 23.6 | 256 | 57.4 | 19.5 | 23.0 | 120 | 28.3 | 17.5 | 54.2 | **<0.001** | **<0.001** |
| TZP | 76 | 88.2 | 1.3 | 10.5 | 225 | 81.3 | 1.8 | 16.9 | 78 | 55.1 | 2.6 | 42.3 | **<0.001** | **<0.001** |
| FEP | 85 | 84.7 | 0.0 | 15.3 | 264 | 78.0 | 1.9 | 20.1 | 119 | 38.7 | 12.6 | 48.7 | **<0.001** | **<0.001** |
| IPM | 44 | 79.5 | 0.0 | 20.5 | 208 | 79.3 | 0.0 | 20.7 | 78 | 48.7 | 0.0 | 51.3 | **<0.001** | **<0.001** |
| MEM | 89 | 85.4 | 0.0 | 14.6 | 264 | 76.9 | 0.4 | 22.7 | 122 | 45.9 | 0.8 | 53.3 | **<0.001** | **<0.001** |
| GEN | 72 | 69.4 | 12.5 | 18.1 | 255 | 54.9 | 4.7 | 40.4 | 101 | 28.7 | 6.9 | 64.4 | **<0.001** | **<0.001** |
| CIP | 86 | 88.4 | 0.0 | 11.6 | 261 | 78.5 | 0.0 | 21.5 | 122 | 47.5 | 0.0 | 52.5 |  | **<0.001** |
| ***P. aeruginosa*** | | | | | | | | | | | | | | |
| TZP | 235 | 23.8 | 4.3 | 71.9 | 367 | 30.2 | 6.5 | 63.2 | 289 | 16.3 | 4.5 | 79.2 | **<0.001** | **0.024** |
| CAZ | 182 | 23.6 | 6.0 | 70.3 | 209 | 28.7 | 2.4 | 68.9 | 172 | 14.5 | 0.6 | 84.9 | **0.003** | **0.022** |
| FEP | 226 | 19.9 | 8.4 | 71.7 | 416 | 22.1 | 5.0 | 72.8 | 232 | 13.8 | 6.5 | 79.7 | **0.040** | 0.071 |
| IPM | 207 | 43.5 | 0.0 | 56.5 | 349 | 44.7 | 0.9 | 54.4 | 275 | 31.6 | 2.2 | 66.2 | **0.004** | **0.008** |
| MEM | 238 | 32.4 | 7.1 | 60.5 | 419 | 38.4 | 5.7 | 55.8 | 348 | 21.8 | 8.0 | 70.1 | **<0.001** | **0.002** |
| CIP | 256 | 28.1 | 4.7 | 67.2 | 416 | 24.3 | 5.5 | 70.2 | 345 | 22.0 | 2.6 | 75.4 | 0.171 | 0.061 |
| ***E. faecium*** | | | | | | | | | | | | | | |
| AMP | 61 | 63.9 | 0.0 | 36.1 | 110 | 68.2 | 0.0 | 31.8 | 105 | 82.9 | 0.0 | 17.1 | **0.011** | **0.004** |
| CIP | 59 | 55.9 | 10.2 | 33.9 | 108 | 53.7 | 13.9 | 32.4 | 103 | 63.1 | 13.6 | 23.3 | 0.242 | 0.142 |
| LVX | 48 | 54.2 | 6.3 | 39.6 | 100 | 43.0 | 13.0 | 44.0 | 93 | 67.7 | 3.2 | 29.0 | **0.020** | 0.063 |
| ERY | 50 | 82.0 | 16.0 | 2.0 | 103 | 84.5 | 12.6 | 2.9 | 101 | 85.1 | 3.0 | 11.9 | **0.024** | **0.013** |
| LNZ | 56 | 0.0 | 0.0 | 100.0 | 110 | 0.0 | 0.9 | 99.1 | 94 | 0.0 | 0.0 | 100.0 | NA | NA |
| VAN | 58 | 32.8 | 0.0 | 67.2 | 108 | 24.1 | 0.0 | 75.9 | 97 | 58.8 | 0.0 | 41.2 | **<0.001** | **<0.001** |
| ***S. aureus*** | | | | | | | | | | | | | | |
| OXA | 265 | 15.8 | 0.0 | 84.2 | 663 | 18.9 | 0.0 | 81.1 | 486 | 18.3 | 0.0 | 81.7 | 0.556 | 0.498 |
| GEN | 263 | 6.8 | 4.9 | 88.2 | 640 | 5.9 | 4.4 | 89.7 | 169 | 10.7 | 3.0 | 86.4 | 0.111 | 0.235 |
| RIF | 191 | 8.4 | 0.0 | 91.6 | 341 | 1.5 | 0.0 | 98.5 | 197 | 0.0 | 0.0 | 100.0 | **<0.001** | **<0.001** |
| CIP | 264 | 18.9 | 0.0 | 81.1 | 663 | 20.7 | 1.2 | 78.1 | 487 | 18.7 | 2.5 | 78.9 | 0.691 | 0.919 |
| LVX | 197 | 17.3 | 0.0 | 82.7 | 620 | 20.2 | 0.3 | 79.5 | 442 | 19.7 | 0.2 | 80.1 | 0.656 | 0.589 |
| SXT | 262 | 3.1 | 0.0 | 96.9 | 646 | 2.5 | 0.0 | 97.5 | 446 | 4.0 | 0.0 | 96.0 | 0.344 | 0.343 |
| CLI | 264 | 28.4 | 0.0 | 71.6 | 662 | 34.0 | 0.0 | 66.0 | 353 | 37.4 | 0.0 | 62.6 | 0.065 | **0.021** |
| ERY | 263 | 28.5 | 0.0 | 71.5 | 646 | 34.7 | 0.2 | 65.2 | 354 | 37.6 | 1.4 | 61.0 | **0.045** | **0.015** |
| LNZ | 263 | 0.0 | 0.0 | 100.0 | 664 | 0.5 | 0.0 | 99.5 | 353 | 0.6 | 0.0 | 99.4 | 0.503 | 0.285 |
| TCY | 248 | 0.4 | 0.0 | 99.6 | 605 | 4.1 | 0.0 | 95.9 | 430 | 3.7 | 0.2 | 96.0 | **0.017** | **0.046** |

* Percent positive

AMC: Amoxicillin/Clavulanic acid, AMP: Ampicillin, CAZ: Ceftazidime, CIP: Ciprofloxacin, CLI: Clindamycin, CRO: Ceftriaxone, CTX: Cefotaxime, ERY: Erythromycin, ESBL: extended spectrum beta-lactamase, ETP: Ertapenem, FEP: Cefepime, FOX: Cefoxitin, GEN: Gentamicin, IPM: Imipenem, LNZ: Linezolid, LVX: Levofloxacin, MEM: Meropenem, OXA: Oxacillin, SAM: Ampicillin/Sulbactam, SXT: Sulfamethoxazole/Trimethoprim, TZP: Piperacillin/Tazobactam, VAN: Vancomycin, ND: Not Determined.

**Suppl Table 4B. Distribution of resistant, intermediate, and susceptible isolates by period in Lower respiratory tract specimens.**

|  | **Pre-pandemic** | | | | **Pandemic** | | | | **Post-pandemic** | | | |  |  |
| --- | --- | --- | --- | --- | --- | --- | --- | --- | --- | --- | --- | --- | --- | --- |
|  | **n** | **%R** | **%I** | **%S** | **n** | **%R** | **%I** | **%S** | **n** | **%R** | **%I** | **%S** | ***p value*** | ***p for trend*** |
| ***K. pneumoniae*** | | | | | | | | | | | | | | |
| ESBL | 200 | 47.5* |  |  | 414 | 38.4* |  |  | 302 | 45.7* |  |  | **0.047** | 0.953 |
| SAM | 238 | 46.6 | 7.6 | 45.8 | 599 | 38.9 | 7.2 | 53.9 | 422 | 43.1 | 7.6 | 49.3 | 0.073 | 0.637 |
| TZP | 245 | 18.0 | 15.9 | 66.1 | 542 | 13.3 | 9.0 | 77.7 | 319 | 15.7 | 7.8 | 76.5 | 0.091 | 0.293 |
| CAZ | 157 | 44.6 | 0.0 | 55.4 | 337 | 36.2 | 0.6 | 63.2 | 171 | 56.7 | 1.2 | 42.1 | **<0.001** | **0.015** |
| CRO | 244 | 45.9 | 0.0 | 54.1 | 615 | 43.3 | 0.5 | 56.3 | 395 | 49.9 | 0.0 | 50.1 | 0.137 | 0.209 |
| FEP | 264 | 34.1 | 1.9 | 64.0 | 616 | 28.1 | 2.9 | 69.0 | 393 | 39.2 | 5.9 | 55.0 | **<0.001** | **0.026** |
| FOX | 111 | 15.3 | 5.4 | 79.3 | 333 | 11.4 | 1.8 | 86.8 | 59 | 8.5 | 1.7 | 89.8 | 0.309 | 0.130 |
| ETP | 259 | 3.1 | 0.0 | 96.9 | 617 | 7.0 | 0.2 | 92.9 | 393 | 6.1 | 0.5 | 93.4 | 0.082 | 0.170 |
| IPM | 208 | 2.4 | 0.5 | 97.1 | 530 | 5.3 | 0.6 | 94.2 | 249 | 5.6 | 0.4 | 94.0 | 0.194 | 0.122 |
| MEM | 247 | 2.0 | 0.0 | 98.0 | 622 | 5.6 | 0.6 | 93.7 | 371 | 5.4 | 0.5 | 94.1 | 0.067 | 0.086 |
| GEN | 241 | 33.6 | 0.0 | 66.4 | 592 | 28.9 | 0.3 | 70.8 | 309 | 31.4 | 0.0 | 68.6 | 0.397 | 0.655 |
| CIP | 244 | 36.9 | 3.7 | 59.4 | 601 | 39.6 | 4.8 | 55.6 | 433 | 35.8 | 12.5 | 51.7 | 0.683 | 0.596 |
| SXT | 159 | 44.0 | 0.0 | 56.0 | 341 | 43.1 | 0.0 | 56.9 | 210 | 57.6 | 0.0 | 42.4 | **0.002** | **0.005** |
| ***E. cloacae*** | | | | | | | | | | | | | | |
| TZP | 139 | 12.2 | 5.0 | 82.7 | 448 | 16.3 | 3.8 | 79.9 | 159 | 20.8 | 2.5 | 76.7 | 0.167 | 0.059 |
| FEP | 138 | 3.6 | 3.6 | 92.8 | 464 | 1.9 | 5.2 | 92.9 | 199 | 12.1 | 5.0 | 82.9 | **<0.001** | **<0.001** |
| ETP | 139 | 5.8 | 7.2 | 87.1 | 467 | 4.5 | 4.7 | 90.8 | 199 | 9.0 | 6.5 | 84.4 | 0.062 | 0.127 |
| IPM | 132 | 2.3 | 0.8 | 97.0 | 445 | 0.2 | 2.0 | 97.8 | 151 | 1.3 | 0.7 | 98.0 | 0.057 | 0.447 |
| MEM | 140 | 1.4 | 0.0 | 98.6 | 467 | 0.2 | 0.2 | 99.6 | 184 | 2.2 | 0.5 | 97.3 | **0.041** | 0.335 |
| GEN | 140 | 3.6 | 0.7 | 95.7 | 466 | 4.3 | 0.0 | 95.7 | 169 | 18.9 | 0.0 | 81.1 | **<0.001** | **<0.001** |
| CIP | 140 | 2.9 | 2.1 | 95.0 | 447 | 2.9 | 3.4 | 93.7 | 199 | 10.1 | 5.0 | 84.9 | **<0.001** | **0.001** |
| SXT | 120 | 4.2 | 0.0 | 95.8 | 267 | 5.2 | 0.0 | 94.8 | 124 | 14.5 | 0.0 | 85.5 | **0.001** | **0.002** |
| ***A baumannii*** | | | | | | | | | | | | | | |
| SAM | 147 | 80.3 | 6.8 | 12.9 | 388 | 57.0 | 24.7 | 18.3 | 170 | 44.1 | 22.9 | 32.9 | **<0.001** | **<0.001** |
| TZP | 161 | 90.1 | 1.2 | 8.7 | 362 | 86.7 | 0.3 | 13.0 | 147 | 75.5 | 2.0 | 22.4 | **0.001** | **0.001** |
| FEP | 189 | 83.6 | 1.1 | 15.3 | 402 | 82.1 | 2.0 | 15.9 | 183 | 48.6 | 24.0 | 27.3 | **<0.001** | **<0.001** |
| IPM | 103 | 82.5 | 0.0 | 17.5 | 343 | 85.7 | 0.0 | 14.3 | 134 | 74.6 | 0.7 | 24.6 | **0.024** | 0.090 |
| MEM | 186 | 84.4 | 0.0 | 15.6 | 402 | 82.6 | 0.5 | 16.9 | 184 | 71.7 | 2.7 | 25.5 | **0.013** | **0.010** |
| GEN | 152 | 77.0 | 8.6 | 14.5 | 385 | 61.6 | 5.7 | 32.7 | 171 | 46.2 | 12.3 | 41.5 | **<0.001** | **<0.001** |
| CIP | 192 | 90.1 | 0.0 | 9.9 | 393 | 83.7 | 0.3 | 16.0 | 183 | 74.3 | 0.0 | 25.7 | **<0.001** | **<0.001** |
| ***P. aeruginosa*** | | | | | | | | | | | | | | |
| TZP | 286 | 21.0 | 7.3 | 71.7 | 705 | 24.8 | 10.5 | 64.7 | 326 | 24.8 | 8.9 | 66.3 | 0.271 | 0.231 |
| CAZ | 159 | 20.1 | 4.4 | 75.5 | 423 | 29.6 | 4.7 | 65.7 | 183 | 26.8 | 1.1 | 72.1 | 0.063 | 0.277 |
| FEP | 294 | 18.4 | 5.8 | 75.9 | 810 | 20.4 | 9.1 | 70.5 | 484 | 18.2 | 10.1 | 71.7 | 0.501 | 0.963 |
| IPM | 232 | 42.2 | 0.9 | 56.9 | 681 | 42.0 | 2.3 | 55.7 | 308 | 38.3 | 2.3 | 59.4 | 0.528 | 0.387 |
| MEM | 295 | 32.9 | 9.5 | 57.6 | 829 | 36.6 | 5.7 | 57.8 | 552 | 35.9 | 8.3 | 55.8 | 0.728 | 0.491 |
| CIP | 319 | 23.8 | 2.5 | 73.7 | 795 | 25.2 | 4.7 | 70.2 | 548 | 26.6 | 5.1 | 68.2 | 0.511 | 0.247 |
| LVX | 93 | 10.8 | 9.7 | 79.6 | 267 | 30.3 | 7.5 | 62.2 | 109 | 28.4 | 5.5 | 66.1 | **0.001** | **0.011** |
| ***S. aureus*** | | | | | | | | | | | | | | |
| OXA | 183 | 13.1 | 0.0 | 86.9 | 596 | 18.1 | 0.0 | 81.9 | 431 | 10.4 | 0.0 | 89.6 | **0.002** | 0.069 |
| GEN | 177 | 6.2 | 0.6 | 93.2 | 589 | 5.3 | 2.7 | 92.0 | 212 | 8.0 | 0.5 | 91.5 | 0.391 | 0.415 |
| RIF | 110 | 0.0 | 0.0 | 100.0 | 365 | 0.3 | 0.0 | 99.7 | 157 | 0.6 | 0.0 | 99.4 | 0.644 | 0.351 |
| CIP | 183 | 14.2 | 0.0 | 85.8 | 596 | 20.8 | 0.3 | 78.9 | 430 | 11.9 | 1.2 | 87.0 | **0.001** | 0.080 |
| LVX | 78 | 23.1 | 0.0 | 76.9 | 345 | 20.3 | 1.2 | 78.6 | 285 | 13.3 | 0.0 | 86.7 | **0.029** | **0.011** |
| SXT | 177 | 4.5 | 0.0 | 95.5 | 591 | 4.9 | 0.0 | 95.1 | 333 | 3.0 | 0.0 | 97.0 | 0.383 | 0.297 |
| CLI | 182 | 25.8 | 0.0 | 74.2 | 609 | 32.2 | 0.2 | 67.7 | 377 | 20.2 | 0.0 | 79.8 | **<0.001** | **0.018** |
| ERY | 174 | 24.1 | 0.0 | 75.9 | 605 | 31.9 | 0.2 | 67.9 | 378 | 18.5 | 1.6 | 79.9 | **<0.001** | **0.014** |
| LNZ | 180 | 0.0 | 0.0 | 100.0 | 610 | 0.2 | 0.0 | 99.8 | 392 | 0.5 | 0.0 | 99.5 | 0.434 | **<0.001** |
| TCY | 78 | 3.8 | 0.0 | 96.2 | 341 | 2.9 | 0.0 | 97.1 | 258 | 3.5 | 0.4 | 96.1 | 0.883 | 0.954 |

* Percent positive

CAZ: Ceftazidime, CIP: Ciprofloxacin, CLI: Clindamycin, CRO: Ceftriaxone, ERY: Erythromycin, ESBL: extended spectrum beta-lactamase, ETP: Ertapenem, FEP: Cefepime, FOX: Cefoxitin, GEN: Gentamicin, IPM: Imipenem, LNZ: Linezolid, LVX: Levofloxacin, MEM: Meropenem, OXA: Oxacillin, RIF: Rifampicin, SAM: Ampicillin/Sulbactam, SXT: Sulfamethoxazole/Trimethoprim, TCY: Tetracycline, TZP: Piperacillin/Tazobactam, ND: Not Determined.

**Suppl Table 4C. Distribution of resistant, intermediate, and susceptible isolates by period in clinical isolates collected from urine.**

|  | **Pre-pandemic** | | | | **Pandemic** | | | | **Post-pandemic** | | | |  |  |
| --- | --- | --- | --- | --- | --- | --- | --- | --- | --- | --- | --- | --- | --- | --- |
|  | **n** | **%R** | **%I** | **%S** | **n** | **%R** | **%I** | **%S** | **n** | **%R** | **%I** | **%S** | ***p value*** | ***p for trend*** |
| ***E. coli*** | | | | | | | | | | | | | | |
| ESBL | 2,818 | 41.9* |  |  | 4,251 | 45.1* |  |  | 10,292 | 47.7* |  |  | **<0.001** | **0.005** |
| AMP | 4,390 | 73.4 | 1.4 | 25.2 | 6,940 | 74.9 | 1.0 | 24.1 | 3,660 | 72.5 | 1.1 | 26.4 | **0.030** | 0.305 |
| AMC | 972 | 15.2 | 22.4 | 62.3 | 1,013 | 10.7 | 21.2 | 68.1 | 1,515 | 13.4 | 20.7 | 65.9 | **0.005** | 0.224 |
| SAM | 3,737 | 38.1 | 21.6 | 40.3 | 7,355 | 38.8 | 18.2 | 42.9 | 9,795 | 37.6 | 18.1 | 44.3 | **0.027** | **0.008** |
| CAZ | 3,800 | 40.1 | 0.3 | 59.6 | 4,594 | 46.4 | 0.3 | 53.3 | 3,323 | 64.8 | 2.1 | 33.0 | **<0.001** | **<0.001** |
| CRO | 4,831 | 41.4 | 0.3 | 58.3 | 8,350 | 47.1 | 0.1 | 52.9 | 10,909 | 44.4 | 0.1 | 55.5 | **<0.001** | **0.045** |
| CTX | 2,838 | 39.8 | 0.4 | 59.8 | 5,137 | 45.8 | 0.3 | 53.9 | 2,609 | 41.4 | 0.5 | 58.1 | **<0.001** | 0.167 |
| FEP | 4,753 | 29.7 | 1.8 | 68.5 | 8,264 | 32.2 | 4.2 | 63.6 | 9,738 | 33.6 | 5.3 | 61.1 | **<0.001** | **<0.001** |
| ETP | 5,434 | 0.9 | 0.0 | 99.1 | 8,306 | 1.6 | 0.2 | 98.2 | 9,673 | 1.6 | 0.4 | 98.0 | **<0.001** | **0.001** |
| IPM | 1,710 | 0.6 | 0.4 | 99.0 | 2,945 | 1.5 | 0.3 | 98.3 | 4,982 | 0.5 | 0.7 | 98.8 | **<0.001** | 0.070 |
| MEM | 5,222 | 0.4 | 0.1 | 99.5 | 8,339 | 1.3 | 0.0 | 98.7 | 8,775 | 1.3 | 0.1 | 98.6 | **<0.001** | **<0.001** |
| CIP | 4,850 | 59.8 | 4.0 | 36.2 | 8,278 | 60.7 | 6.9 | 32.4 | 11,013 | 56.3 | 16.2 | 27.6 | **<0.001** | **<0.001** |
| ***K. pneumoniae*** | | | | | | | | | | | | | | |
| ESBL | 394 | 40.9* |  |  | 697 | 46.5* |  |  | 1,453 | 41.4* |  |  | 0.061 | 0.536 |
| SAM | 560 | 38.4 | 7.7 | 53.9 | 1,240 | 43.7 | 6.7 | 49.6 | 1,826 | 35.3 | 7.4 | 57.3 | **<0.001** | **0.005** |
| CAZ | 562 | 33.8 | 0.0 | 66.2 | 777 | 40.2 | 0.3 | 59.6 | 618 | 58.9 | 1.3 | 39.8 | **<0.001** | **<0.001** |
| CRO | 691 | 34.9 | 0.1 | 65.0 | 1,377 | 44.2 | 0.0 | 55.8 | 1,940 | 39.9 | 0.1 | 60.0 | **<0.001** | 0.234 |
| CTX | 411 | 33.1 | 0.0 | 66.9 | 854 | 40.7 | 0.0 | 59.3 | 331 | 45.0 | 0.0 | 55.0 | **0.003** | **0.001** |
| FEP | 686 | 27.4 | 0.9 | 71.7 | 1,362 | 29.7 | 2.4 | 67.9 | 1,640 | 35.0 | 3.2 | 61.8 | **<0.001** | **<0.001** |
| ETP | 776 | 1.9 | 0.1 | 97.9 | 1,364 | 4.5 | 0.0 | 95.5 | 1,629 | 2.8 | 0.4 | 96.8 | **0.002** | 0.762 |
| IPM | 243 | 2.1 | 0.0 | 97.9 | 461 | 5.9 | 0.0 | 94.1 | 588 | 1.9 | 1.5 | 96.6 | **0.001** | 0.281 |
| MEM | 738 | 1.6 | 0.0 | 98.4 | 1,371 | 3.7 | 0.0 | 96.3 | 1,459 | 1.8 | 0.4 | 97.8 | **0.001** | 0.584 |
| CIP | 698 | 37.4 | 3.6 | 59.0 | 1,362 | 44.2 | 5.5 | 50.3 | 1,967 | 38.4 | 9.8 | 51.8 | **0.002** | 0.448 |
| ***P. aeruginosa*** | | | | | | | | | | | | | | |
| TZP | 246 | 18.7 | 9.3 | 72.0 | 377 | 23.4 | 5.8 | 70.8 | 578 | 22.3 | 4.7 | 73.0 | 0.513 | 0.549 |
| CAZ | 223 | 15.7 | 1.8 | 82.5 | 349 | 20.6 | 2.3 | 77.1 | 311 | 18.0 | 1.3 | 80.7 | 0.301 | 0.616 |
| FEP | 294 | 19.7 | 1.7 | 78.6 | 696 | 25.1 | 2.9 | 72.0 | 689 | 17.6 | 7.1 | 75.3 | **0.006** | 0.234 |
| IPM | 136 | 25.7 | 1.5 | 72.8 | 266 | 31.6 | 3.4 | 65.0 | 532 | 29.1 | 6.0 | 64.8 | 0.403 | 0.463 |
| MEM | 319 | 23.5 | 1.9 | 74.6 | 692 | 28.3 | 2.9 | 68.8 | 825 | 22.4 | 4.6 | 73.0 | **0.035** | 0.380 |
| AMK | 334 | 20.1 | 1.8 | 78.1 | 704 | 23.0 | 2.0 | 75.0 | 645 | 20.6 | 1.6 | 77.8 | 0.415 | 0.921 |
| CIP | 345 | 29.9 | 4.1 | 66.1 | 690 | 30.6 | 4.8 | 64.6 | 821 | 30.5 | 3.9 | 65.7 | 0.950 | 0.912 |
| NOR | 153 | 20.3 | 4.6 | 75.2 | 414 | 24.9 | 3.6 | 71.5 | 343 | 29.4 | 3.2 | 67.3 | 0.094 | 0.030 |
| ***E. faecium*** | | | | | | | | | | | | | | |
| AMP | 113 | 76.1 | 0.0 | 23.9 | 218 | 83.9 | 0.0 | 16.1 | 216 | 86.1 | 0.0 | 13.9 | 0.065 | 0.029 |
| GEH | 96 | 18.8 | 0.0 | 81.3 | 211 | 18.5 | 0.0 | 81.5 | 200 | 22.5 | 0.0 | 77.5 | 0.557 | 0.360 |
| CIP | 111 | 58.6 | 15.3 | 26.1 | 223 | 54.3 | 21.1 | 24.7 | 214 | 63.6 | 14.5 | 22.0 | 0.457 | 0.289 |
| LVX | 75 | 54.7 | 10.7 | 34.7 | 195 | 49.7 | 9.7 | 40.5 | 203 | 62.1 | 5.4 | 32.5 | 0.119 | 0.210 |
| ERY | 90 | 81.1 | 16.7 | 2.2 | 204 | 91.2 | 7.8 | 1.0 | 149 | 88.6 | 6.7 | 4.7 | 0.094 | 0.163 |
| NIT | 99 | 22.2 | 57.6 | 20.2 | 214 | 19.6 | 60.3 | 20.1 | 199 | 42.2 | 36.2 | 21.6 | **0.037** | **0.033** |
| LNZ | 97 | 0.0 | 0.0 | 100.0 | 214 | 1.4 | 0.9 | 97.7 | 202 | 1.0 | 1.0 | 98.0 | 0.504 | 0.547 |
| VAN | 110 | 32.7 | 0.0 | 67.3 | 215 | 17.7 | 0.5 | 81.9 | 203 | 44.3 | 0.0 | 55.7 | **<0.001** | **0.002** |
| TCY | 77 | 48.1 | 0.0 | 51.9 | 187 | 72.2 | 0.0 | 27.8 | 169 | 49.7 | 0.0 | 50.3 | **<0.001** | 0.318 |

* Percent positive

|  |  |
| --- | --- |

AMC: Amoxicillin/Clavulanic acid, AMK: Amikacin, AMP: Ampicillin, CAZ: Ceftazidime, CIP: Ciprofloxacin, CRO: Ceftriaxone, CTX: Cefotaxime, ERY: Erythromycin, ESBL: extended spectrum beta-lactamase, ETP: Ertapenem, FEP: Cefepime, GEH: high-dose gentamicin, IPM: Imipenem, LNZ: Linezolid, LVX: Levofloxacin, MEM: Meropenem, NIT: Nitrofurantoin, NOR: Norfloxacin, SAM: Ampicillin/Sulbactam, TCY: Tetracycline, TZP: Piperacillin/Tazobactam, VAN: Vancomycin, ND: Not Determined.

**Suppl Table 4D. Distribution of resistant, intermediate, and susceptible isolates by period in clinical isolates recovered from abscesses and biopsies.**

|  | Pre-pandemic | | | | Pandemic | | | | Post-pandemic | | | |  |  |
| --- | --- | --- | --- | --- | --- | --- | --- | --- | --- | --- | --- | --- | --- | --- |
|  | **n** | **%R** | **%I** | **%S** | **n** | **%R** | **%I** | **%S** | **n** | **%R** | **%I** | **%S** | ***p value*** | ***p for trend*** |
| ***E. coli*** | | | | | | | | | | | | | | |
| ESBL | 228 | 56.6* |  |  | 452 | 64.4* |  |  | 470 | 63.0* |  |  | 0.129 | 0.185 |
| AMP | 162 | 81.5 | 0.0 | 18.5 | 165 | 86.7 | 2.4 | 10.9 | 108 | 88.0 | 1.9 | 10.2 | 0.081 | **0.042** |
| SAM | 455 | 45.9 | 22.6 | 31.4 | 1,123 | 45.3 | 16.8 | 37.8 | 1,017 | 48.8 | 15.3 | 35.9 | 0.208 | 0.961 |
| CAZ | 456 | 59.9 | 0.0 | 40.1 | 760 | 60.1 | 0.1 | 39.7 | 529 | 79.6 | 2.6 | 17.8 | **<0.001** | **<0.001** |
| CRO | 541 | 61.4 | 0.2 | 38.4 | 1,147 | 64.5 | 0.1 | 35.4 | 1,014 | 65.7 | 0.2 | 34.1 | 0.235 | 0.103 |
| FEP | 486 | 54.5 | 1.9 | 43.6 | 1,137 | 49.1 | 2.8 | 48.1 | 893 | 62.3 | 5.2 | 32.6 | **<0.001** | **<0.001** |
| FOX | 485 | 18.8 | 5.4 | 75.9 | 941 | 19.1 | 6.0 | 74.9 | 94 | 19.1 | 8.5 | 72.3 | 0.961 | 0.779 |
| ETP | 554 | 3.8 | 0.2 | 96.0 | 1,140 | 6.2 | 0.2 | 93.6 | 895 | 11.2 | 0.7 | 88.2 | **<0.001** | **<0.001** |
| IPM | 482 | 2.5 | 0.4 | 97.1 | 1,004 | 5.1 | 0.4 | 94.5 | 403 | 11.4 | 2.5 | 86.1 | **<0.001** | **<0.001** |
| MEM | 545 | 2.2 | 0.0 | 97.8 | 1,141 | 5.2 | 0.4 | 94.5 | 646 | 9.6 | 0.5 | 89.9 | **<0.001** | **<0.001** |
| GEN | 533 | 34.1 | 0.8 | 65.1 | 1,118 | 28.0 | 0.7 | 71.3 | 582 | 34.0 | 0.2 | 65.8 | **0.009** | 0.988 |
| CIP | 540 | 68.3 | 4.1 | 27.6 | 1,135 | 67.5 | 6.0 | 26.5 | 1,058 | 63.5 | 15.8 | 20.7 | 0.119 | 0.060 |
| SXT | 169 | 59.2 | 0.0 | 40.8 | 235 | 66.4 | 0.4 | 33.2 | 244 | 63.5 | 0.0 | 36.5 | 0.304 | 0.447 |
| ***K. pneumoniae*** | | | | | | | | | | | | | | |
| ESBL | 84 | 48.8* |  |  | 225 | 52.4* |  |  | 164 | 60.4* |  |  | 0.153 | 0.060 |
| SAM | 137 | 42.3 | 4.4 | 53.3 | 392 | 45.4 | 7.1 | 47.4 | 367 | 43.9 | 7.4 | 48.8 | 0.659 | 0.722 |
| TZP | 158 | 7.0 | 17.1 | 75.9 | 339 | 20.1 | 10.9 | 69.0 | 279 | 16.8 | 6.8 | 76.3 | **0.002** | 0.090 |
| CAZ | 137 | 42.3 | 0.0 | 57.7 | 221 | 44.8 | 0.0 | 55.2 | 157 | 72.6 | 0.6 | 26.8 | **<0.001** | **<0.001** |
| CRO | 160 | 43.1 | 0.0 | 56.9 | 393 | 48.6 | 0.0 | 51.4 | 356 | 50.6 | 0.6 | 48.9 | 0.268 | 0.120 |
| FEP | 143 | 35.7 | 0.7 | 63.6 | 391 | 30.4 | 0.8 | 68.8 | 274 | 54.0 | 3.6 | 42.3 | **<0.001** | **<0.001** |
| ETP | 163 | 1.8 | 0.6 | 97.5 | 394 | 7.4 | 0.3 | 92.4 | 276 | 5.8 | 1.1 | 93.1 | **0.040** | 0.171 |
| IPM | 152 | 1.3 | 1.3 | 97.4 | 364 | 4.7 | 1.9 | 93.4 | 135 | 7.4 | 4.4 | 88.1 | **0.036** | **0.010** |
| MEM | 162 | 1.9 | 0.0 | 98.1 | 394 | 4.3 | 0.5 | 95.2 | 204 | 4.9 | 1.5 | 93.6 | 0.276 | 0.142 |
| GEN | 160 | 26.2 | 0.0 | 73.8 | 390 | 29.2 | 0.5 | 70.3 | 179 | 28.5 | 0.0 | 71.5 | 0.761 | 0.666 |
| CIP | 160 | 37.5 | 8.1 | 54.4 | 392 | 42.9 | 7.1 | 50.0 | 371 | 44.2 | 8.9 | 46.9 | 0.294 | 0.131 |
| ***P. aeruginosa*** | | | | | | | | | | | | | | |
| TZP | 217 | 26.3 | 8.8 | 65.0 | 442 | 32.1 | 12.9 | 55.0 | 387 | 26.9 | 5.9 | 67.2 | **0.028** | 0.570 |
| CAZ | 177 | 24.3 | 6.2 | 69.5 | 207 | 35.3 | 7.2 | 57.5 | 244 | 25.4 | 2.0 | 72.5 | **0.011** | 0.761 |
| FEP | 202 | 20.8 | 8.4 | 70.8 | 475 | 25.7 | 8.8 | 65.5 | 349 | 18.9 | 13.5 | 67.6 | 0.108 | 0.577 |
| IPM | 184 | 47.3 | 0.0 | 52.7 | 433 | 48.5 | 0.2 | 51.3 | 374 | 43.0 | 1.9 | 55.1 | 0.399 | 0.321 |
| MEM | 195 | 39.5 | 6.7 | 53.8 | 471 | 41.8 | 6.6 | 51.6 | 471 | 32.3 | 10.8 | 56.9 | **0.034** | 0.053 |
| CIP | 225 | 32.4 | 4.4 | 63.1 | 472 | 30.7 | 5.7 | 63.6 | 469 | 27.7 | 4.7 | 67.6 | 0.358 | 0.166 |
| ***E. faecium*** | | | | | | | | | | | | | | |
| CIP | 138 | 47.8 | 20.3 | 31.9 | 159 | 42.1 | 18.9 | 39.0 | 198 | 45.5 | 19.2 | 35.4 | 0.454 | 0.625 |
| LVX | 119 | 48.7 | 8.4 | 42.9 | 158 | 42.4 | 7.6 | 50.0 | 198 | 43.4 | 10.1 | 46.5 | 0.507 | 0.498 |
| ERY | 135 | 71.1 | 23.0 | 5.9 | 157 | 75.2 | 16.6 | 8.3 | 196 | 61.2 | 30.6 | 8.2 | 0.580 | 0.298 |
| LNZ | 142 | 0.0 | 0.0 | 100.0 | 165 | 1.8 | 0.6 | 97.6 | 156 | 0.0 | 0.6 | 99.4 | 0.065 | 0.951 |
| VAN | 146 | 19.2 | 0.0 | 80.8 | 168 | 16.1 | 0.0 | 83.9 | 160 | 41.9 | 0.0 | 58.1 | **<0.001** | **<0.001** |
| ***S. aureus*** | | | | | | | | | | | | | | |
| OXA | 218 | 12.8 | 0.0 | 87.2 | 524 | 23.5 | 0.0 | 76.5 | 498 | 19.5 | 0.0 | 80.5 | **0.004** | 0.203 |
| GEN | 217 | 7.4 | 2.3 | 90.3 | 520 | 8.5 | 4.6 | 86.9 | 159 | 12.6 | 1.9 | 85.5 | 0.204 | 0.097 |
| RIF | 185 | 0.5 | 0.0 | 99.5 | 283 | 1.1 | 0.7 | 98.2 | 258 | 1.9 | 0.0 | 98.1 | 0.401 | 0.181 |
| CIP | 221 | 16.7 | 1.8 | 81.4 | 526 | 25.3 | 1.1 | 73.6 | 500 | 21.8 | 1.2 | 77.0 | **0.038** | 0.387 |
| LVX | 172 | 16.9 | 0.6 | 82.6 | 468 | 25.9 | 0.9 | 73.3 | 476 | 22.1 | 0.2 | 77.7 | **0.045** | 0.537 |
| SXT | 214 | 3.7 | 0.0 | 96.3 | 528 | 5.7 | 0.0 | 94.3 | 456 | 5.3 | 0.0 | 94.7 | 0.553 | 0.529 |
| CLI | 220 | 29.5 | 0.0 | 70.5 | 525 | 41.3 | 0.0 | 58.7 | 335 | 36.4 | 0.0 | 63.6 | **0.009** | 0.215 |
| ERY | 217 | 27.2 | 0.0 | 72.8 | 527 | 38.7 | 0.6 | 60.7 | 336 | 36.3 | 2.4 | 61.3 | **0.009** | 0.038 |
| LNZ | 220 | 0.0 | 0.0 | 100.0 | 527 | 0.2 | 0.0 | 99.8 | 353 | 1.1 | 0.0 | 98.9 | 0.067 | 0.032 |
| TCY | 199 | 5.0 | 0.0 | 95.0 | 464 | 3.2 | 0.0 | 96.8 | 447 | 3.8 | 0.0 | 96.2 | 0.541 | 0.607 |

* Percent positive

AMP: Ampicillin, CAZ: Ceftazidime, CIP: Ciprofloxacin, CLI: Clindamycin, CRO: Ceftriaxone, ERY: Erythromycin, ESBL: extended spectrum beta-lactamase, ETP: Ertapenem, FEP: Cefepime, FOX: Cefoxitin, GEN: Gentamicin, IPM: Imipenem, LNZ: Linezolid, LVX: Levofloxacin, MEM: Meropenem, OXA: Oxacillin, RIF: Rifampicin, SAM: Ampicillin/Sulbactam, SXT: Sulfamethoxazole/Trimethoprim, TCY: Tetracycline, TZP: Piperacillin/Tazobactam, VAN: Vancomycin, ND: Not Determined.

**Supplementary Table 5. Temporal trends in antimicrobial resistance (pre- vs post-pandemic, all isolates).**

|  | Pre-pandemic %R (n) | Δ (%) | Post-pandemic %R (n) | OR | -95% CI | +95% CI |
| --- | --- | --- | --- | --- | --- | --- |
| E. coli |  |  |  |  |  |  |
| ESBL | 46.5 (3,924) | +2.4 | 48.9 (13,299) | 0.906 | 0.844 | 0.973 |
| AMP | 75.1 (5,236) | +0.8 | 75.9 (5,052) | 0.964 | 0.880 | 1.057 |
| AMC | 16.3 (1,230) | −2.1 | 14.2 (1,588) | 1.217 | 0.984 | 1.505 |
| SAM | 41.3 (5,602) | −1.6 | 39.7 (14,804) | 1.199 | 1.119 | 1.285 |
| CAZ | 44.7 (5,263) | +19.3 | 64.0 (5,090) | 0.423 | 0.391 | 0.459 |
| CRO | 47.2 (7,009) | +1.6 | 48.8 (14,703) | 0.939 | 0.887 | 0.995 |
| CTX | 40.4 (3,032) | +4.9 | 45.3 (3,209) | 0.813 | 0.736 | 0.900 |
| FEP | 34.1 (6,907) | +4.3 | 38.4 (14,554) | 0.777 | 0.731 | 0.825 |
| FOX | 13.1 (3,134) | +6.6 | 19.7 (3,860) | 0.603 | 0.529 | 0.688 |
| ETP | 2.0 (7,818) | +1.2 | 3.2 (14,461) | 0.620 | 0.516 | 0.745 |
| IPM | 2.1 (3,484) | +0.2 | 2.3 (6,615) | 0.876 | 0.660 | 1.162 |
| MEM | 1.2 (7,518) | +1.5 | 2.7 (13,048) | 0.452 | 0.359 | 0.570 |
| CIP | 61.3 (7,040) | −3.2 | 58.1 (16,275) | 0.774 | 0.728 | 0.824 |
| SXT | 52.9 (5,232) | +3.4 | 56.3 (12,628) | 0.875 | 0.820 | 0.933 |
| K. pneumoniae |  |  |  |  |  |  |
| ESBL | 45.9 (1,066) | −1.9 | 44.0 (2,785) | 1.078 | 0.935 | 1.242 |
| SAM | 44.5 (1,455) | −6.3 | 38.2 (3,794) | 1.298 | 1.145 | 1.472 |
| CAZ | 37.8 (1,146) | +16.6 | 54.4 (1,317) | 0.493 | 0.419 | 0.580 |
| CRO | 42.1 (1,655) | +1.5 | 43.6 (3,620) | 0.940 | 0.835 | 1.057 |
| FEP | 28.5 (1,711) | +7.8 | 36.3 (3,387) | 0.672 | 0.592 | 0.763 |
| ETP | 3.8 (1,842) | +0.3 | 4.1 (3,380) | 0.932 | 0.695 | 1.250 |
| IPM | 3.6 (1,021) | +0.9 | 4.5 (1,471) | 0.792 | 0.525 | 1.194 |
| MEM | 3.6 (1,755) | −0.2 | 3.4 (3,056) | 1.064 | 0.773 | 1.464 |
| CIP | 37.4 (1,676) | +0.3 | 37.7 (3,993) | 0.906 | 0.803 | 1.022 |
| SXT | 43.9 (1,078) | +4.5 | 48.4 (2,452) | 0.832 | 0.720 | 0.961 |
| K. aerogenes |  |  |  |  |  |  |
| TZP | 20.4 (54) | +19.1 | 39.5 (43) | 0.391 | 0.159 | 0.964 |
| FEP | 4.1 (74) | +5.4 | 9.5 (168) | 0.404 | 0.114 | 1.433 |
| ETP | 1.3 (75) | +2.9 | 4.2 (167) | 0.305 | 0.037 | 2.525 |
| IPM | 0.0 (45) | — | 6.9 (29) | 0.000 | - | - |
| MEM | 0.0 (79) | — | 6.4 (141) | 0.000 | - | - |
| GEN | 3.8 (79) | +8.0 | 11.8 (93) | 0.291 | 0.078 | 1.082 |
| CIP | 8.9 (79) | +3.4 | 12.3 (171) | 0.653 | 0.265 | 1.608 |
| E. cloacae |  |  |  |  |  |  |
| FEP | 5.1 (432) | +5.0 | 10.1 (1,146) | 0.469 | 0.293 | 0.751 |
| ETP | 4.4 (454) | +4.9 | 9.3 (1,152) | 0.449 | 0.275 | 0.734 |
| IPM | 2.9 (374) | +0.6 | 3.5 (821) | 0.811 | 0.401 | 1.642 |
| MEM | 2.4 (462) | +2.8 | 5.2 (961) | 0.441 | 0.227 | 0.855 |
| CIP | 7.6 (462) | +7.8 | 15.4 (1,160) | 0.429 | 0.293 | 0.628 |
| SXT | 7.5 (292) | +28.0 | 35.5 (932) | 0.148 | 0.094 | 0.233 |
| A. baumannii |  |  |  |  |  |  |
| SAM | 71.5 (442) | −30.8 | 40.7 (653) | 3.549 | 2.633 | 4.784 |
| TZP | 86.2 (457) | −16.5 | 69.7 (422) | 2.748 | 1.945 | 3.884 |
| FEP | 82.2 (552) | −33.7 | 48.5 (691) | 3.081 | 2.330 | 4.075 |
| IPM | 77.2 (298) | −13.3 | 63.9 (374) | 1.896 | 1.342 | 2.678 |
| MEM | 82.5 (550) | −17.4 | 65.1 (700) | 2.527 | 1.923 | 3.319 |
| AMK | 51.9 (131) | −13.4 | 38.5 (379) | 1.530 | 1.023 | 2.290 |
| GEN | 72.9 (457) | −26.4 | 46.5 (620) | 3.174 | 2.389 | 4.216 |
| CIP | 86.8 (559) | −18.9 | 67.9 (692) | 3.082 | 2.301 | 4.128 |
| LVX | 52.3 (65) | +2.8 | 55.1 (49) | 0.894 | 0.425 | 1.881 |
| SXT | 80.3 (223) | −17.1 | 63.2 (337) | 2.423 | 1.625 | 3.614 |
| P. aeruginosa |  |  |  |  |  |  |
| TZP | 21.1 (1,556) | +0.3 | 21.4 (2,200) | 0.987 | 0.841 | 1.159 |
| CAZ | 18.8 (969) | +1.2 | 20.0 (1,138) | 0.954 | 0.768 | 1.186 |
| FEP | 18.2 (1,626) | −2.6 | 15.6 (2,955) | 1.172 | 0.997 | 1.378 |
| IPM | 35.8 (1,223) | −3.4 | 32.4 (2,060) | 1.124 | 0.967 | 1.305 |
| MEM | 29.9 (1,685) | −4.1 | 25.8 (3,457) | 1.195 | 1.048 | 1.362 |
| CIP | 28.1 (1,820) | −2.1 | 26.0 (3,437) | 1.123 | 0.987 | 1.277 |
| LVX | 18.1 (353) | +12.1 | 30.2 (348) | 0.513 | 0.358 | 0.734 |
| E. faecium |  |  |  |  |  |  |
| AMP | 66.6 (398) | +7.0 | 73.6 (686) | 0.714 | 0.546 | 0.934 |
| CIP | 56.0 (373) | −1.3 | 54.7 (649) | 1.008 | 0.753 | 1.351 |
| LVX | 55.8 (303) | −1.5 | 54.3 (608) | 1.038 | 0.775 | 1.391 |
| ERY | 78.0 (332) | −3.1 | 74.9 (589) | 2.078 | 1.102 | 3.919 |
| LNZ | 0.3 (367) | +0.4 | 0.7 (607) | 0.411 | 0.046 | 3.695 |
| VAN | 29.1 (388) | +14.0 | 43.1 (610) | 0.542 | 0.413 | 0.711 |
| S. aureus |  |  |  |  |  |  |
| OXA | 13.9 (1,427) | +2.6 | 16.5 (3,549) | 0.816 | 0.686 | 0.972 |
| FOX | 10.2 (246) | −5.2 | 5.0 (239) | 2.140 | 1.049 | 4.365 |
| GEN | 9.5 (1,526) | +6.9 | 16.4 (1,362) | 0.533 | 0.426 | 0.667 |
| CIP | 16.1 (1,569) | +2.3 | 18.4 (3,604) | 0.853 | 0.727 | 1.000 |
| LVX | 15.9 (1,185) | +3.3 | 19.2 (3,248) | 0.798 | 0.668 | 0.954 |
| SXT | 5.1 (1,493) | +1.3 | 6.4 (3,016) | 0.789 | 0.600 | 1.037 |
| CLI | 27.8 (1,503) | +3.3 | 31.1 (2,764) | 0.855 | 0.744 | 0.982 |
| ERY | 29.7 (1,417) | +1.1 | 30.8 (2,753) | 0.937 | 0.814 | 1.078 |

OR: odds ratio (post vs pre period); CI: confidence interval (95%); Δ: absolute change in resistance (%)

“-” indicates estimates not calculated due to zero or extremely low counts

**Supplementary Table 6. Temporal trends in antimicrobial resistance (pre- vs post-pandemic, in isolates recovered from blood).**

|  | Pre-pandemic %R (n) | Δ (%) | Post-pandemic %R (n) | OR | -95% CI | +95% CI |
| --- | --- | --- | --- | --- | --- | --- |
| E. coli |  |  |  |  |  |  |
| ESBL | 53.8 (117) | +10.3 | 64.1 (410) | 0.652 | 0.430 | 0.988 |
| AMP | 84.9 (186) | +3.0 | 87.9 (99) | 0.778 | 0.377 | 1.607 |
| SAM | 43.3 (545) | −0.7 | 42.6 (1083) | 1.386 | 1.089 | 1.764 |
| CAZ | 51.6 (587) | +2.0 | 53.6 (735) | 0.899 | 0.723 | 1.118 |
| CRO | 53.4 (674) | +12.0 | 65.4 (557) | 0.608 | 0.483 | 0.766 |
| FEP | 47.9 (566) | +15.0 | 62.9 (857) | 0.482 | 0.387 | 0.601 |
| FOX | 16.0 (619) | −1.7 | 14.3 (133) | 1.171 | 0.688 | 1.994 |
| DOR | 5.7 (35) | −2.6 | 3.1 (32) | 1.818 | 0.157 | 21.088 |
| ETP | 7.4 (688) | +2.8 | 10.2 (863) | 0.712 | 0.496 | 1.021 |
| IPM | 5.7 (610) | +9.2 | 14.9 (442) | 0.346 | 0.225 | 0.533 |
| MEM | 5.1 (680) | +6.7 | 11.8 (620) | 0.403 | 0.265 | 0.612 |
| CIP | 60.6 (673) | −2.7 | 57.9 (1098) | 0.514 | 0.411 | 0.643 |
| SXT | 59.5 (185) | +0.9 | 60.4 (202) | 0.962 | 0.640 | 1.445 |
| K. pneumoniae |  |  |  |  |  |  |
| ESBL | 43.0 (86) | +13.4 | 56.4 (218) | 0.583 | 0.352 | 0.965 |
| SAM | 46.3 (203) | −5.3 | 41.0 (456) | 1.141 | 0.811 | 1.606 |
| CRO | 46.3 (227) | +8.3 | 54.6 (284) | 0.716 | 0.505 | 1.017 |
| FEP | 28.9 (218) | +15.1 | 44.0 (357) | 0.507 | 0.353 | 0.729 |
| ETP | 10.9 (238) | −3.1 | 7.8 (358) | 1.448 | 0.826 | 2.538 |
| IPM | 9.9 (182) | −1.4 | 8.5 (211) | 1.153 | 0.581 | 2.291 |
| MEM | 10.4 (230) | −2.0 | 8.4 (311) | 1.283 | 0.716 | 2.299 |
| CIP | 34.8 (227) | +1.3 | 36.1 (462) | 0.878 | 0.624 | 1.235 |
| SXT | 43.9 (66) | +21.9 | 65.8 (111) | 0.408 | 0.218 | 0.762 |
| E. cloacae |  |  |  |  |  |  |
| FEP | 1.4 (70) | +5.6 | 7.0 (258) | 0.197 | 0.026 | 1.499 |
| ETP | 2.7 (75) | +3.8 | 6.5 (263) | 0.408 | 0.092 | 1.811 |
| IPM | 1.6 (62) | +2.9 | 4.5 (112) | 0.321 | 0.037 | 2.816 |
| MEM | 1.3 (75) | +3.0 | 4.3 (185) | 0.296 | 0.036 | 2.406 |
| CIP | 8.0 (75) | +3.2 | 11.2 (267) | 0.641 | 0.256 | 1.605 |
| A. baumannii |  |  |  |  |  |  |
| SAM | 70.8 (72) | −42.5 | 28.3 (120) | 5.735 | 2.882 | 11.412 |
| TZP | 88.2 (76) | −33.1 | 55.1 (78) | 6.427 | 2.714 | 15.221 |
| FEP | 84.7 (85) | −46.0 | 38.7 (119) | 6.983 | 3.447 | 14.149 |
| IPM | 79.5 (44) | −30.8 | 48.7 (78) | 4.094 | 1.738 | 9.641 |
| MEM | 85.4 (89) | −39.5 | 45.9 (122) | 6.786 | 3.409 | 13.506 |
| GEN | 69.4 (72) | −40.7 | 28.7 (101) | 8.621 | 4.068 | 18.267 |
| CIP | 88.4 (86) | −40.9 | 47.5 (122) | 8.386 | 3.965 | 17.735 |
| P. aeruginosa |  |  |  |  |  |  |
| TZP | 23.8 (235) | −7.5 | 16.3 (289) | 1.615 | 1.044 | 2.496 |
| CAZ | 23.6 (182) | −9.1 | 14.5 (172) | 1.962 | 1.135 | 3.391 |
| FEP | 19.9 (226) | −6.1 | 13.8 (232) | 1.606 | 0.974 | 2.647 |
| IPM | 43.5 (207) | −11.9 | 31.6 (275) | 1.609 | 1.106 | 2.342 |
| MEM | 32.4 (238) | −10.6 | 21.8 (348) | 1.717 | 1.176 | 2.505 |
| CIP | 28.1 (256) | −6.1 | 22.0 (345) | 1.432 | 0.984 | 2.085 |
| E. faecium |  |  |  |  |  |  |
| AMP | 63.9 (61) | +19.0 | 82.9 (105) | 0.367 | 0.177 | 0.760 |
| CIP | 55.9 (59) | +7.2 | 63.1 (103) | 0.609 | 0.295 | 1.260 |
| LVX | 54.2 (48) | +13.5 | 67.7 (93) | 0.586 | 0.279 | 1.234 |
| ERY | 82.0 (50) | +3.1 | 85.1 (101) | 5.721 | 0.719 | 45.504 |
| LNZ | 0.0 | - | 0.0 | - | - | - |
| VAN | 32.8 (58) | +26.0 | 58.8 (97) | 0.342 | 0.173 | 0.676 |
| S. aureus |  |  |  |  |  |  |
| OXA | 15.8 (265) | +2.5 | 18.3 (486) | 0.840 | 0.562 | 1.256 |
| GEN | 6.8 (263) | +3.9 | 10.7 (169) | 0.629 | 0.317 | 1.249 |
| RIF | 8.4 (191) | −8.4 | 0.0 (197) | - | - | - |
| CIP | 18.9 (264) | −0.2 | 18.7 (487) | 0.986 | 0.672 | 1.447 |
| LVX | 17.3 (197) | +2.4 | 19.7 (442) | 0.849 | 0.548 | 1.315 |
| SXT | 3.1 (262) | +0.9 | 4.0 (446) | 0.749 | 0.321 | 1.747 |
| CLI | 28.4 (264) | +9.0 | 37.4 (353) | 0.664 | 0.471 | 0.937 |
| ERY | 28.5 (263) | +9.1 | 37.6 (354) | 0.648 | 0.459 | 0.914 |
| TCY | 0.4 (248) | +3.3 | 3.7 (430) | 0.105 | 0.014 | 0.793 |

OR: odds ratio (post vs pre period); CI: confidence interval (95%); Δ: absolute change in resistance (%)

“-” indicates estimates not calculated due to zero or extremely low counts

**Supplementary Table 7. Temporal trends in antimicrobial resistance (pre- vs post-pandemic, in lower respiratory isolates).**

|  | Pre-pandemic %R (n) | Δ (%) | Post-pandemic %R (n) | OR | -95% CI | +95% CI |
| --- | --- | --- | --- | --- | --- | --- |
| K. pneumoniae |  |  |  |  |  |  |
| ESBL | 47.5 (200) | −1.8 | 45.7 (302) | 1.075 | 0.752 | 1.538 |
| SAM | 46.6 (238) | −3.5 | 43.1 (422) | 1.164 | 0.836 | 1.620 |
| TZP | 18.0 (245) | −2.3 | 15.7 (319) | 1.325 | 0.844 | 2.081 |
| CAZ | 44.6 (157) | +12.1 | 56.7 (171) | 0.597 | 0.385 | 0.926 |
| CRO | 45.9 (244) | +4.0 | 49.9 (395) | 0.853 | 0.619 | 1.174 |
| FEP | 34.1 (264) | +5.1 | 39.2 (393) | 0.747 | 0.538 | 1.038 |
| FOX | 15.3 (111) | −6.8 | 8.5 (59) | 2.048 | 0.714 | 5.874 |
| ETP | 3.1 (259) | +3.0 | 6.1 (393) | 0.487 | 0.215 | 1.102 |
| IPM | 2.4 (208) | +3.2 | 5.6 (249) | 0.414 | 0.146 | 1.169 |
| MEM | 2.0 (247) | +3.4 | 5.4 (371) | 0.361 | 0.133 | 0.974 |
| GEN | 33.6 (241) | −2.2 | 31.4 (309) | 1.106 | 0.772 | 1.585 |
| CIP | 36.9 (244) | −1.1 | 35.8 (433) | 0.897 | 0.643 | 1.252 |
| SXT | 44.0 (159) | +13.6 | 57.6 (210) | 0.579 | 0.382 | 0.877 |
| E. cloacae |  |  |  |  |  |  |
| TZP | 12.2 (139) | +8.6 | 20.8 (159) | 0.547 | 0.289 | 1.035 |
| FEP | 3.6 (138) | +8.5 | 12.1 (199) | 0.269 | 0.100 | 0.723 |
| ETP | 5.8 (139) | +3.2 | 9.0 (199) | 0.617 | 0.260 | 1.466 |
| IPM | 2.3 (132) | −1.0 | 1.3 (151) | 1.734 | 0.285 | 10.543 |
| MEM | 1.4 (140) | +0.8 | 2.2 (184) | 0.649 | 0.117 | 3.593 |
| GEN | 3.6 (140) | +15.3 | 18.9 (169) | 0.160 | 0.060 | 0.422 |
| CIP | 2.9 (140) | +7.2 | 10.1 (199) | 0.254 | 0.085 | 0.761 |
| SXT | 4.2 (120) | +10.3 | 14.5 (124) | 0.256 | 0.092 | 0.714 |
| - 1. baumannii |  |  |  |  |  |  |
| SAM | 80.3 (147) | −36.2 | 44.1 (170) | 4.637 | 2.557 | 8.411 |
| TZP | 90.1 (161) | −14.6 | 75.5 (147) | 3.079 | 1.572 | 6.031 |
| FEP | 83.6 (189) | −35.0 | 48.6 (183) | 3.061 | 1.809 | 5.180 |
| IPM | 82.5 (103) | −7.9 | 74.6 (134) | 1.558 | 0.819 | 2.964 |
| MEM | 84.4 (186) | −12.7 | 71.7 (184) | 1.928 | 1.149 | 3.234 |
| AMK | 65.2 (46) | −26.5 | 38.7 (106) | 2.744 | 1.329 | 5.666 |
| GEN | 77.0 (152) | −30.8 | 46.2 (171) | 4.780 | 2.739 | 8.342 |
| CIP | 90.1 (192) | −15.8 | 74.3 (183) | 3.147 | 1.765 | 5.610 |
| SXT | 76.0 (75) | −22.6 | 53.4 (58) | 2.758 | 1.316 | 5.778 |
| P. aeruginosa |  |  |  |  |  |  |
| TZP | 21.0 (286) | +3.8 | 24.8 (326) | 0.780 | 0.531 | 1.147 |
| CAZ | 20.1 (159) | +6.7 | 26.8 (183) | 0.718 | 0.432 | 1.196 |
| FEP | 18.4 (294) | −0.2 | 18.2 (484) | 0.955 | 0.654 | 1.394 |
| IPM | 42.2 (232) | −3.9 | 38.3 (308) | 1.151 | 0.812 | 1.632 |
| MEM | 32.9 (295) | +3.0 | 35.9 (552) | 0.888 | 0.653 | 1.206 |
| CIP | 23.8 (319) | +2.8 | 26.6 (548) | 0.828 | 0.601 | 1.143 |
| LVX | 10.8 (93) | +17.6 | 28.4 (109) | 0.314 | 0.143 | 0.687 |
| S. aureus |  |  |  |  |  |  |
| OXA | 13.1 (183) | −2.7 | 10.4 (431) | 1.295 | 0.763 | 2.197 |
| GEN | 6.2 (177) | +1.8 | 8.0 (212) | 0.761 | 0.347 | 1.670 |
| RIF | 0.0 (110) | +0.6 | 0.6 (157) | 0.000 | - | - |
| CIP | 14.2 (183) | −2.3 | 11.9 (430) | 1.214 | 0.731 | 2.018 |
| LVX | 23.1 (78) | −9.8 | 13.3 (285) | 1.950 | 1.041 | 3.653 |
| SXT | 4.5 (177) | −1.5 | 3.0 (333) | 1.529 | 0.592 | 3.946 |
| CLI | 25.8 (182) | −5.6 | 20.2 (377) | 1.379 | 0.909 | 2.091 |
| ERY | 24.1 (174) | −5.6 | 18.5 (378) | 1.373 | 0.890 | 2.118 |
| LNZ | 0.0 | - | 0.5 | 0.000 | - | - |
| TCY | 3.8 (78) | −0.3 | 3.5 (258) | 1.102 | 0.291 | 4.176 |

OR: odds ratio (post vs pre period); CI: confidence interval (95%); Δ: absolute change in resistance (%)

“-” indicates estimates not calculated due to zero or extremely low counts

**Supplementary Table 8. Temporal trends in antimicrobial resistance (pre- vs post-pandemic, in urine isolates).**

|  | Pre-pandemic %R (n) | Δ (%) | Post-pandemic %R (n) | OR | -95% CI | +95% CI |
| --- | --- | --- | --- | --- | --- | --- |
| E. coli |  |  |  |  |  |  |
| ESBL | 41.9 (2818) | +5.8 | 47.7 (10292) | 0.79 | 0.73 | 0.86 |
| AMP | 73.4 (4390) | −0.9 | 72.5 (3660) | 1.06 | 0.96 | 1.17 |
| AMC | 15.2 (972) | −1.8 | 13.4 (1515) | 1.20 | 0.95 | 1.52 |
| SAM | 38.1 (3737) | −0.5 | 37.6 (9795) | 1.11 | 1.02 | 1.21 |
| CAZ | 40.1 (3800) | +24.7 | 64.8 (3323) | 0.34 | 0.31 | 0.38 |
| CRO | 41.4 (4831) | +3.0 | 44.4 (10909) | 0.89 | 0.83 | 0.95 |
| CTX | 39.8 (2838) | +1.6 | 41.4 (2609) | 0.93 | 0.84 | 1.04 |
| FEP | 29.7 (4753) | +3.9 | 33.6 (9738) | 0.79 | 0.73 | 0.85 |
| ETP | 0.9 (5434) | +0.7 | 1.6 (9673) | 0.54 | 0.39 | 0.76 |
| IPM | 0.6 (1710) | −0.1 | 0.5 (4982) | 1.21 | 0.58 | 2.54 |
| MEM | 0.4 (5222) | +0.9 | 1.3 (8775) | 0.32 | 0.20 | 0.51 |
| CIP | 59.8 (4850) | −3.5 | 56.3 (11013) | 0.81 | 0.75 | 0.87 |
| K. pneumoniae |  |  |  |  |  |  |
| ESBL | 40.9 (394) | +0.5 | 41.4 (1453) | 0.98 | 0.78 | 1.22 |
| SAM | 38.4 (560) | −3.1 | 35.3 (1826) | 1.15 | 0.94 | 1.41 |
| CAZ | 33.8 (562) | +25.1 | 58.9 (618) | 0.35 | 0.27 | 0.44 |
| CRO | 34.9 (691) | +5.0 | 39.9 (1940) | 0.81 | 0.67 | 0.97 |
| CTX | 33.1 (411) | +11.9 | 45.0 (331) | 0.60 | 0.45 | 0.81 |
| FEP | 27.4 (686) | +7.6 | 35.0 (1640) | 0.67 | 0.55 | 0.82 |
| ETP | 1.9 (776) | +0.9 | 2.8 (1629) | 0.69 | 0.38 | 1.25 |
| IPM | 2.1 (243) | −0.2 | 1.9 (588) | 1.08 | 0.37 | 3.16 |
| MEM | 1.6 (738) | +0.2 | 1.8 (1459) | 0.91 | 0.46 | 1.81 |
| CIP | 37.4 (698) | +1.0 | 38.4 (1967) | 0.85 | 0.71 | 1.02 |
| E. cloacae |  |  |  |  |  |  |
| FEP | 12.3 (73) | +0.3 | 12.6 (230) | 0.97 | 0.44 | 2.16 |
| ETP | 4.7 (85) | +6.2 | 10.9 (230) | 0.39 | 0.13 | 1.16 |
| IPM | 4.5 (44) | −1.0 | 3.5 (370) | 1.23 | 0.27 | 5.64 |
| MEM | 2.3 (87) | +6.9 | 9.2 (173) | 0.23 | 0.05 | 1.03 |
| CIP | 12.6 (87) | +7.7 | 20.3 (232) | 0.54 | 0.27 | 1.11 |
| NOR | 4.8 (42) | +9.1 | 13.9 (122) | 0.31 | 0.07 | 1.39 |
| SXT | 13.4 (82) | +35.5 | 48.9 (509) | 0.16 | 0.08 | 0.31 |
| A. baumannii |  |  |  |  |  |  |
| SAM | 76.7 (43) | −36.7 | 40.0 (50) | 8.25 | 2.72 | 25.02 |
| FEP | 87.5 (56) | −51.7 | 35.8 (53) | 7.31 | 2.50 | 21.32 |
| MEM | 89.3 (56) | −26.3 | 63.0 (54) | 4.90 | 1.78 | 13.47 |
| GEN | 80.0 (45) | −28.2 | 51.8 (56) | 3.26 | 1.26 | 8.43 |
| CIP | 92.7 (55) | −24.2 | 68.5 (54) | 5.86 | 1.82 | 18.85 |
| P. aeruginosa |  |  |  |  |  |  |
| TZP | 18.7 (246) | +3.6 | 22.3 (578) | 0.85 | 0.58 | 1.24 |
| CAZ | 15.7 (223) | +2.3 | 18.0 (311) | 0.85 | 0.54 | 1.35 |
| FEP | 19.7 (294) | −2.1 | 17.6 (689) | 1.08 | 0.76 | 1.53 |
| IPM | 25.7 (136) | +3.4 | 29.1 (532) | 0.79 | 0.51 | 1.21 |
| MEM | 23.5 (319) | −1.1 | 22.4 (825) | 1.03 | 0.75 | 1.39 |
| AMK | 20.1 (334) | +0.5 | 20.6 (645) | 0.97 | 0.70 | 1.35 |
| CIP | 29.9 (345) | +0.6 | 30.5 (821) | 0.97 | 0.74 | 1.28 |
| NOR | 20.3 (153) | +9.1 | 29.4 (343) | 0.62 | 0.39 | 0.98 |
| E. faecium |  |  |  |  |  |  |
| AMP | 76.1 (113) | +10.0 | 86.1 (216) | 0.51 | 0.29 | 0.92 |
| GEH | 18.8 (96) | +3.7 | 22.5 (200) | 0.79 | 0.43 | 1.46 |
| CIP | 58.6 (111) | +5.0 | 63.6 (214) | 0.77 | 0.45 | 1.34 |
| LVX | 54.7 (75) | +7.4 | 62.1 (203) | 0.83 | 0.46 | 1.47 |
| ERY | 81.1 (90) | +7.5 | 88.6 (149) | 1.94 | 0.39 | 9.56 |
| NIT | 22.2 (99) | +20.0 | 42.2 (199) | 0.56 | 0.28 | 1.14 |
| LNZ | 0.0 (97) | +1.0 | 1.0 (202) | 0.00 | - | - |
| VAN | 32.7 (110) | +11.6 | 44.3 (203) | 0.61 | 0.38 | 0.99 |
| TCY | 48.1 (77) | +1.6 | 49.7 (169) | 0.94 | 0.55 | 1.61 |
| S. aureus |  |  |  |  |  |  |
| GEN | 2.8 (71) | +37.6 | 40.4 (270) | 0.04 | 0.01 | 0.17 |
| RIF | 0.0 (51) | +1.7 | 1.7 (60) | 0.00 | - | - |
| CIP | 16.7 (72) | +2.7 | 19.4 (160) | 0.83 | 0.40 | 1.72 |
| LVX | 22.9 (48) | −1.6 | 21.3 (150) | 1.09 | 0.50 | 2.37 |
| SXT | 3.9 (51) | +16.2 | 20.1 (354) | 0.16 | 0.04 | 0.69 |
| CLI | 25.7 (35) | +6.9 | 32.6 (95) | 0.71 | 0.30 | 1.71 |
| ERY | 29.4 (34) | +5.3 | 34.7 (95) | 0.80 | 0.34 | 1.89 |
| NIT | 0.0 (72) | 0.0 | 0.0 (134) | - | - | - |
| LNZ | 0.0 (72) | +0.8 | 0.8 (127) | 0.00 | - | - |
| TCY | 9.1 (55) | −4.1 | 5.0 (139) | 1.89 | 0.57 | 6.22 |

OR: odds ratio (post vs pre period); CI: confidence interval (95%); Δ: absolute change in resistance (%)

“-” indicates estimates not calculated due to zero or extremely low counts

**Supplementary Table 9. Temporal trends in antimicrobial resistance (pre- vs post-pandemic, all isolates, and by period and in clinical isolates recovered from biopsies and abscesses).**

|  | Pre-pandemic %R (n) | Δ (%) | Post-pandemic %R (n) | OR | -95% CI | +95% CI |
| --- | --- | --- | --- | --- | --- | --- |
| E. coli |  |  |  |  |  |  |
| ESBL | 63.0 (470) | +6.4 | 56.6 (228) | 0.77 | 0.56 | 1.06 |
| AMP | 88.0 (108) | +6.5 | 81.5 (162) | 0.51 | 0.24 | 1.07 |
| SAM | 48.8 (1017) | +2.9 | 45.9 (455) | 1.08 | 0.84 | 1.38 |
| CAZ | 79.6 (529) | +19.7 | 59.9 (456) | 0.33 | 0.25 | 0.45 |
| CRO | 65.7 (1014) | +4.3 | 61.4 (541) | 0.83 | 0.67 | 1.03 |
| FEP | 62.3 (893) | +7.8 | 54.5 (486) | 0.65 | 0.52 | 0.82 |
| FOX | 19.1 (94) | +0.3 | 18.8 (485) | 0.93 | 0.53 | 1.65 |
| ETP | 11.2 (895) | +7.4 | 3.8 (554) | 0.31 | 0.19 | 0.50 |
| IPM | 11.4 (403) | +8.9 | 2.5 (482) | 0.19 | 0.10 | 0.37 |
| MEM | 9.6 (646) | +7.4 | 2.2 (545) | 0.21 | 0.11 | 0.40 |
| GEN | 34.0 (582) | −0.1 | 34.1 (533) | 1.01 | 0.79 | 1.30 |
| CIP | 63.5 (1058) | −4.8 | 68.3 (540) | 0.81 | 0.63 | 1.03 |
| SXT | 63.5 (244) | +4.3 | 59.2 (169) | 0.83 | 0.56 | 1.24 |
| K. pneumoniae |  |  |  |  |  |  |
| ESBL | 60.4 (164) | +11.6 | 48.8 (84) | 0.63 | 0.37 | 1.06 |
| SAM | 43.9 (367) | +1.6 | 42.3 (137) | 0.88 | 0.59 | 1.32 |
| TZP | 16.8 (279) | +9.8 | 7.0 (158) | 0.42 | 0.21 | 0.83 |
| CAZ | 72.6 (157) | +30.3 | 42.3 (137) | 0.27 | 0.17 | 0.44 |
| CRO | 50.6 (356) | +7.5 | 43.1 (160) | 0.73 | 0.50 | 1.07 |
| FEP | 54.0 (274) | +18.3 | 35.7 (143) | 0.44 | 0.29 | 0.67 |
| ETP | 5.8 (276) | +4.0 | 1.8 (163) | 0.30 | 0.09 | 1.06 |
| IPM | 7.4 (135) | +6.1 | 1.3 (152) | 0.16 | 0.03 | 0.75 |
| MEM | 4.9 (204) | +3.0 | 1.9 (162) | 0.36 | 0.10 | 1.33 |
| GEN | 28.5 (179) | +2.3 | 26.2 (160) | 0.89 | 0.55 | 1.44 |
| CIP | 44.2 (371) | +6.7 | 37.5 (160) | 0.73 | 0.49 | 1.08 |
| SXT | 64.1 (103) | +6.4 | 57.7 (52) | 0.76 | 0.39 | 1.51 |
| P. aeruginosa |  |  |  |  |  |  |
| TZP | 26.9 (387) | +0.6 | 26.3 (217) | 1.01 | 0.69 | 1.48 |
| CAZ | 25.4 (244) | +1.1 | 24.3 (177) | 1.00 | 0.64 | 1.57 |
| FEP | 18.9 (349) | −1.9 | 20.8 (202) | 1.05 | 0.68 | 1.63 |
| IPM | 43.0 (374) | −4.3 | 47.3 (184) | 1.15 | 0.80 | 1.64 |
| MEM | 32.3 (471) | −7.2 | 39.5 (195) | 1.29 | 0.91 | 1.84 |
| CIP | 27.7 (469) | −4.7 | 32.4 (225) | 1.25 | 0.88 | 1.78 |
| E. faecium |  |  |  |  |  |  |
| CIP | 45.5 (198) | −2.3 | 47.8 (138) | 1.17 | 0.71 | 1.91 |
| LVX | 43.4 (198) | −5.3 | 48.7 (119) | 1.22 | 0.75 | 1.96 |
| ERY | 61.2 (196) | −9.9 | 71.1 (135) | 1.60 | 0.66 | 3.90 |
| LNZ | 0.0 | — | 0.0 | - | - | - |
| VAN | 41.9 (160) | +22.7 | 19.2 (146) | 0.33 | 0.20 | 0.55 |
| S. aureus |  |  |  |  |  |  |
| OXA | 19.5 (498) | +6.7 | 12.8 (218) | 0.61 | 0.39 | 0.96 |
| GEN | 12.6 (159) | +5.2 | 7.4 (217) | 0.56 | 0.28 | 1.11 |
| RIF | 1.9 (258) | +1.4 | 0.5 (185) | 0.28 | 0.03 | 2.37 |
| CIP | 21.8 (500) | +5.1 | 16.7 (221) | 0.73 | 0.48 | 1.10 |
| LVX | 22.1 (476) | +5.2 | 16.9 (172) | 0.72 | 0.46 | 1.13 |
| SXT | 5.3 (456) | +1.6 | 3.7 (214) | 0.70 | 0.31 | 1.58 |
| CLI | 36.4 (335) | +6.9 | 29.5 (220) | 0.73 | 0.51 | 1.05 |
| ERY | 36.3 (336) | +9.1 | 27.2 (217) | 0.63 | 0.43 | 0.92 |
| LNZ | 1.1 | — | 0.0 | 0.00 | - | - |
| TCY | 3.8 (447) | −1.2 | 5.0 (199) | 1.34 | 0.60 | 2.98 |

OR: odds ratio (post vs pre period); CI: confidence interval (95%); Δ: absolute change in resistance (%)

“-” indicates estimates not calculated due to zero or extremely low counts
